# Cycloaddition Strategies for the Synthesis of Diverse Heterocyclic Spirocycles for Fragment‐Based Drug Discovery

**DOI:** 10.1002/ejoc.201900847

**Published:** 2019-07-29

**Authors:** Thomas A. King, Hannah L. Stewart, Kim T. Mortensen, Andrew J. P. North, Hannah F. Sore, David R. Spring

**Affiliations:** ^1^ Department of Chemistry University of Cambridge Lensfield Road 1EW Cambridge CB21EW

**Keywords:** Cycloaddition, Diversity‐oriented synthesis, Heterocycles, Spirocycles, Fragment‐based drug discovery

## Abstract

In recent years the pharmaceutical industry has benefited from the advances made in fragment‐based drug discovery (FBDD) with more than 30 fragment‐derived drugs currently marketed or progressing through clinical trials. The success of fragment‐based drug discovery is entirely dependent upon the composition of the fragment screening libraries used. Heterocycles are prevalent within marketed drugs due to the role they play in providing binding interactions; consequently, heterocyclic fragments are important components of FBDD libraries. Current screening libraries are dominated by flat, sp^2^‐rich compounds, primarily owing to their synthetic tractability, despite the superior physicochemical properties displayed by more three‐dimensional scaffolds. Herein, we report step‐efficient routes to a number of biologically relevant, fragment‐like heterocyclic spirocycles. The use of both electron‐deficient and electron‐rich 2‐atom donors was explored in complexity‐generating [3+2]‐cycloadditions to furnish products in 3 steps from commercially available starting materials. The resulting compounds were primed for further fragment elaboration through the inclusion of synthetic handles from the outset of the syntheses.

## Introduction

Heterocycles play a vital role in drug discovery. The incorporated heteroatoms provide potential binding interactions and the cyclic nature limits structural flexibility, thereby reducing the entropic penalty of binding^1^, ^2^ Heterocycles are prevalent in marketed drugs both as flat heteroaromatics and as saturated, fused or spirocyclic scaffolds. However, this variety is not representative of typical screening libraries, which are dominated by flat heteroaromatic scaffolds as opposed to more synthetically‐challenging, three‐dimensional, saturated, heterocyclic scaffolds.^2^

The failure of high throughput screening (HTS) to provide the expected plethora of new drugs with the rise of combinatorial chemistry can be strongly attributed to the dominance of sp^2^‐rich, flat compounds in HTS libraries.^3^, ^4^, ^5^ This realisation has led to the development of new strategies and approaches towards drug discovery, including, but by no means limited to, diversity‐oriented synthesis^6^, ^7^ (DOS) and fragment‐based drug discovery^8^, ^9^, ^10^ (FBDD). FBDD was developed as a means of efficiently sampling chemical space by limiting the size (typically <300 kDa) and physicochemical properties of screening compounds.^8^, ^10^, ^11^ Fragment hits, as a result of their small size, typically have weak but high‐quality binding interactions,^11^ with the potential for linking, merging and growing into high‐affinity drug‐like candidates without the physical property inflation which is a common affliction in the development of candidates identified through more traditional methods.^12^ As the FBDD field has evolved, the focus has turned to the importance of the fragment library composition. Unfortunately, as with HTS, the robust chemistry surrounding aromatic ring systems has led to a significant over‐representation of flat, sp^2^‐rich scaffolds.^13^, ^14^

In 2016, a key publication by Astex highlighted that the various approaches to drug discovery, for example, DOS and FBDD, might not be considered orthogonal.^14^ In fact, DOS might be applied to the construction of more diverse and three‐dimensional fragment screening libraries. DOS is focused on using short and divergent synthetic sequences from simple starting materials to generate structurally complex and diverse scaffolds.^15^, ^16^, ^17^, ^18^, ^19^, ^20^, ^21^, ^22^ DOS would provide the ideal strategy to expand fragment libraries into three‐dimensional chemical space, and this challenge has been taken up across industry and academia.^23^, ^24^, ^25^, ^26^, ^27^, ^28^, ^29^

Within three‐dimensional fragment space, spirocycles are an important class of saturated ring systems.^30^, ^31^ The quaternary centre provides an inherent three‐dimensionality to this sub‐set of scaffolds whilst their cyclic nature provides all of the benefits of reduced flexibility. It has been shown that these properties are largely responsible for the biological activity observed with many spirocyclic compounds since they are linked to improved physicochemical properties and decreased entropic binding penalties.^32^, ^33^

**Figure 1:**
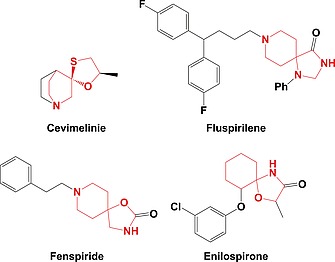
Examples of spirocycle‐containing marketed drugs.^30^, ^31^

Despite almost 47,000 biologically active compounds, including a number of marketed drugs (Figure 1), containing a spirocyclic scaffold, spirocycles are under‐represented in fragment screening libraries.^33^ This is likely due to the synthetic challenge posed by the generation of the quaternary centre. Regio‐ and stereo‐control is difficult to achieve and, historically, has required multiple alkylation steps followed by intramolecular coupling. Furthermore, these routes have suffered from functional group incompatibility which, in turn, has greatly limited the incorporation of functional handles for late‐stage modification and fragment elaboration.^34^, ^35^, ^36^ Moreover, these strategies tend to involve long linear sequences, and are therefore of limited use in the optimisation of fragment hits.^34^, ^35^, ^36^

**Figure 2:**
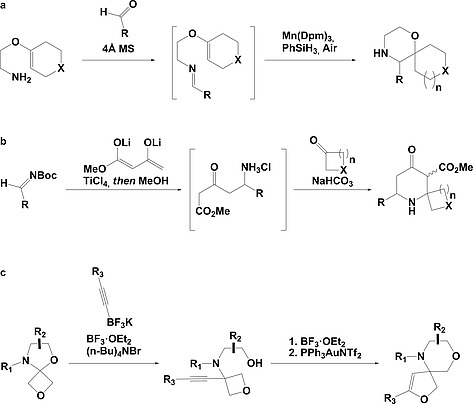
Recent advances in the synthesis of heterocyclic spirocycles (a) Wang and Bode generated unactivated imines and utilised metal hydride hydrogen atom transfer chemistry with manganese to generate a C‐centered radical, which underwent addition to the unactivated imine to generate the spirocycle with an N‐centered radical from which the unprotected‐N was unveiled by a second hydrogen atom transfer: 19 spirocycles with R = alkyl or aryl; X = NCbz, S or CH_2_; *n* = 5 or 6;^46^ (b) Griggs et al. formed highly substituted 2‐spiropiperidines in a one‐pot reaction from *N*‐Boc imines via the addition of Chan's diene under Maitland–Japp conditions followed by in‐situ Boc‐deprotection and a sodium bicarbonate catalysed cyclisation onto a cyclic ketone: 10 spirocycles with R = alkyl or aryl; X = NCbz, O, S or CH_2_; *n* = 4, 5 or 6;^47^ (c) Brady and Carreira used the addition of trifluoroborates to oxetanyl *N*,*O*‐acetals to generate products with oxetane and alkyne functionalities from which spiro heterocycles can be generated by orthogonal activation, such as intramolecular opening of the oxetane by the alcohol followed by catalytic intramolecular alkyne hydroalkoxylation to close the spirocyclic ring: 5 spirocycles with R = alkyl, aryl or TMS.^42^

Despite this, due to their biological potential, there has been a recent surge of interest in the synthesis of spirocycles for drug discovery.^26^, ^30^, ^37^, ^38^, ^39^, ^40^, ^41^, ^42^, ^43^, ^44^, ^45^ In particular, Wang and Bode utilised metal hydride hydrogen atom transfer chemistry to generate saturated heterocyclic spirocycles in a one‐pot reaction from unactivated imines (Figure 2a).^46^ Work by Griggs et al. also used a one‐pot reaction, this time to generate highly substituted 2‐spiropiperidines from *N*‐Boc imines, by in‐situ Boc‐deprotection and cyclisation onto a cyclic ketone (Figure 2b).^47^ Alternatively, the addition of trifluoroborates to oxetanyl *N*,*O*‐acetals by Carreira and co‐workers provided compounds for spirocyclisation via orthogonal activation of the oxetane and alkyne functionalities (Figure 2c).^42^

There is a growing need for the development of step‐efficient and versatile strategies for the synthesis of these compounds. Heterocyclic, spirocyclic scaffolds, in particular, represent an underexplored and biologically relevant area of chemical space.

Herein, we report the application of DOS‐inspired methodology to synthesise a diverse range of heterocyclic, spirocyclic scaffolds. A key [3+2]‐cycloaddition step has been utilised to provide high levels of regio‐selectivity and improve previously lengthy procedures to generate the core quaternary carbon centre. Furthermore, synthetic handles for late‐stage elaboration were incorporated from the outset to aid FBDD hit exploration.

## Results and Discussion

**Figure 3:**
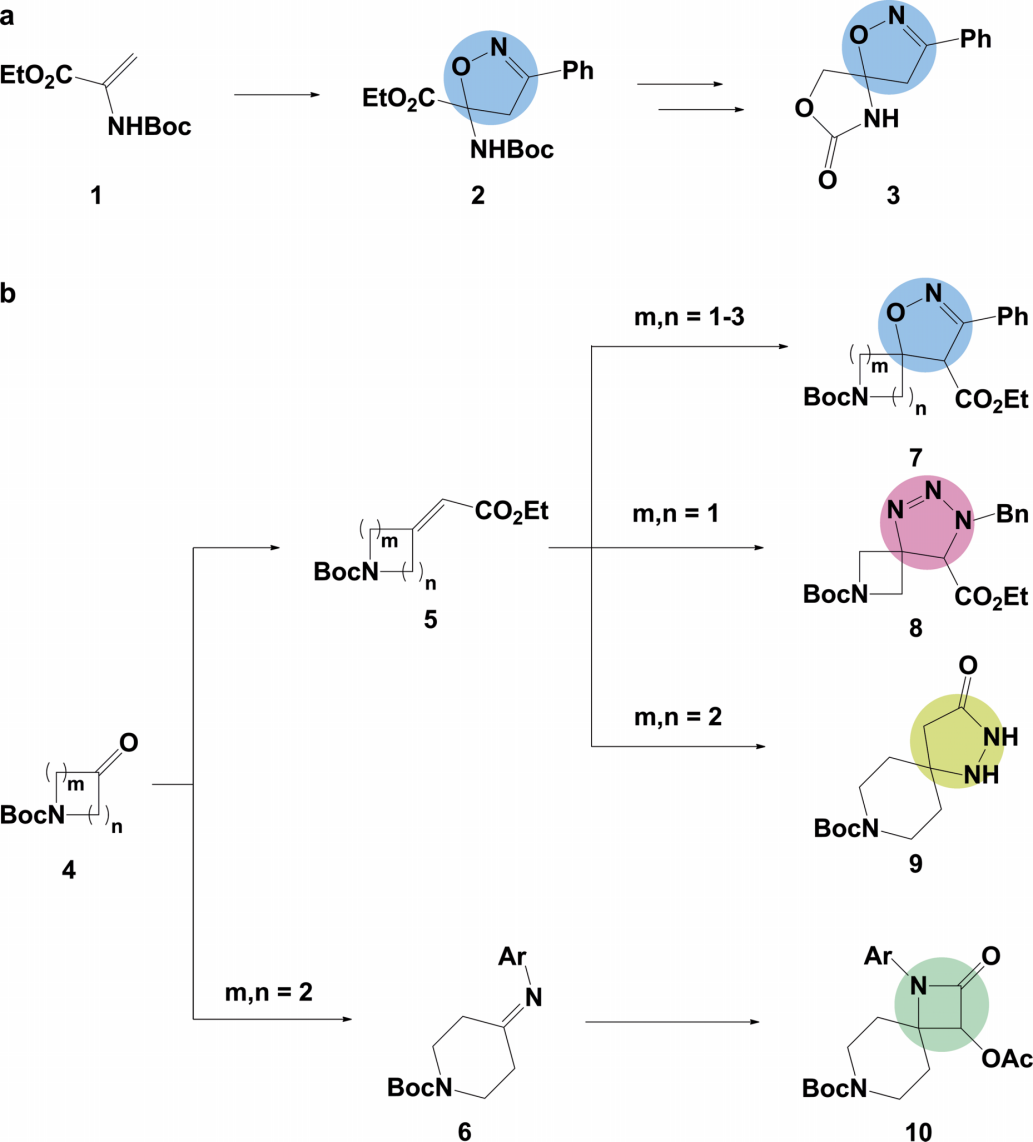
The use of both acyclic (a) and exo‐cyclic (b) starting materials allowed the efficient synthesis of heterocyclic spirocyclic compounds. Based on the cyclisation approach taken, different functional handles in the final compounds [for example, ester (blue and pink), carbonyl (yellow and green), and aromatic (pink and green) moieties] were available for further modifications.

We envisaged that [3+2] cycloadditions could afford a range of heterocyclic, spirocyclic scaffolds from simple starting materials in just a few steps (Figure 3). Acyclic, electron‐rich alkenes could be reacted to introduce a heterocyclic moiety which, provided the starting material was selected appropriately, could then be formed into a spirocycle by the coupling of functional groups on the lower half of the molecule (Figure 3a). Alternatively, exo‐cyclic, electron‐deficient alkenes might offer a means to introduce a quaternary carbon centre directly (Figure 3b). These later compounds could also offer alternative routes to spirocycle formation.

In keeping with the goal of generating diverse scaffolds, we aimed to carry out [3+2]‐cycloadditions on both acyclic, electron‐rich (**1**) and exo‐cyclic, electron‐deficient (**5**) alkenes. Our initial studies focused on the cycloadditions of nitrile‐oxide dipole **13** with both the acyclic alkene and exo‐cyclic alkene (Figure 3). Spirocycle **3** could be built after the key cycloaddition produced isoxazole **2**, whereas spirocycle **7** could be formed during the key cycloaddition step on a saturated heterocycle (**5** or **6**).

**Scheme 1:**
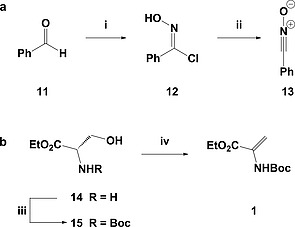
Formation of key dipole (a) and acyclic (b) starting materials: (i) **11** (1.0 equiv.), NH_2_OH (2.0 equiv.), EtOH, r.t., 10 min; then NCS (1.1 equiv.), DMF, r.t., 0.5 h, 99 %; (ii) **12** (1.0 equiv.), NEt_3_ (1.0 equiv.), DCE, 0 °C, 3 min, used immediately; (iii) **14** (1.0 equiv.), Boc_2_O (0.95 equiv.), K_2_CO_3_ (1.5 equiv.), EtOAc, H_2_O, r.t., 12 h, 93 %; (iv) **15** (1.0 equiv.), MsCl (1.25 equiv.), NEt_3_ (3.0 equiv.), DCM, –15 °C ‐ r.t., 2 h, 94 %.

Each building block was synthesised in 1 or 2 steps from inexpensive, commercially available starting materials (Scheme 1, Table 1). Treatment of benzaldehyde (**11**) with hydroxylamine to give the corresponding oxime, followed by addition of *N*‐chlorosuccinimide, proceeded smoothly to yield chlorooxime **12** in an excellent 99 % yield (Scheme 1a).^48^ This was used directly, generating **13** in situ, or converted to dipole **13** before addition.

**Table 1:**
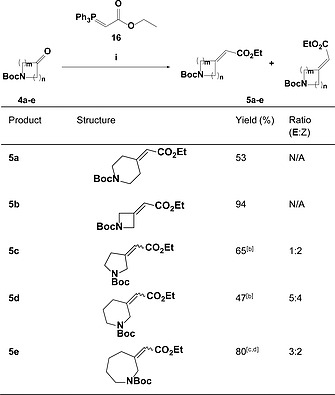
Generation of electron‐deficient alkenes for [3+2]‐cycloadditions^a^

a Reaction conditions: (i) **4** (1.0 equiv.), **16** (1.1 equiv.), DCM, reflux, 6–44 h^51^

Notes: Yield corresponding to both isomers combined.

Yield corresponding to the inseparable mixture of both isomers.

5 equiv. of **16** used.

We imagined that 1,1‐disubstituted alkenes such as **1** could act as suitable [3+2]‐cycloaddition partners. The functional groups present provided the synthetic handles necessary for cyclisation to form the second ring. As such, following work by Ramesh et al., serine ethyl ester **14** was first Boc‐protected before an elimination reaction using mesyl chloride and triethylamine provided the desired 1,1‐disubstituted alkene **1** in an excellent yield (Scheme 1b).^49^ The amino acid starting material provided both an amine and ester as the synthetic handles required for cyclisation of the second ring, with the potential for a range of cyclisation strategies.

The exo‐cyclic alkene cycloaddition partners were synthesised from a range of commercially available Boc‐protected *N*‐heterocyclic ketones. Heating with Wittig reagent **16** generated electron‐deficient alkenes of general structure **5** in good to excellent yields (Table 1). As might be expected, the yields obtained closely followed the trend in ring strain; the 4‐ and 7‐membered cycles reacted with the highest yields, whilst the relatively unstrained 6‐membered rings proceeded least effectively.^50^ For symmetrical ketones **4a** and **4b** the reaction proceeded smoothly to yield the 6‐ and 4‐membered heterocycles **5a** and **5b** in 94 % and 53 % respectively. For asymmetrical ketones **4c** and **4d** the reaction led to a separable mixture of stereoisomers. The major isomers from these reactions, isolated in 41 % and 26 % yield respectively, were carried forward for the subsequent cyclisation. For asymmetrical ketone **4e**, the increased flexibility afforded by the 7‐membered ring reduced the chromatographic separation of the two isomers (*E/Z*) such that they were carried forward as a mixture in the ratio 3:2. Critically, these intermediates contained both an amine and an ester moiety enabling opportunities for further fragment elaboration.

**Scheme 2:**
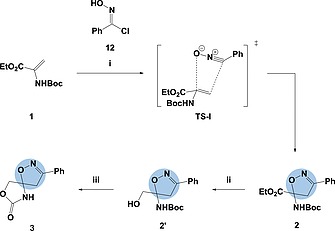
Synthesis of isoxazole‐based spirocycle **3**, via [3+2]‐cycloaddition with nitrile‐oxide dipole generated in situ, followed by carbamate formation: (i) **1** (1.0 equiv.), **12** (6.0 equiv.) NEt_3_ (6.8 equiv.), DCM, r.t., 1 h, 58 %; (ii) **2** (1.0 equiv.), NaBH_4_ (1.0 equiv.), CH_3_OH/THF (1:8), 0 °C ‐ r.t., 12 h, 55 %; (iii) **2′** (1.0 equiv.), KO*t*Bu (2.0 equiv.), THF, 0 °C, 1 h, 56 %.

The [3+2]‐cycloaddition was initially tested on acyclic, electron‐rich alkene **1** (Scheme 2). The desired nitrile‐oxide dipole was generated in situ from previously prepared chlorooxime (**12**) using triethylamine in dichloromethane.^52^, ^53^ Pleasingly, this provided isoxazole heterocycle **2** in a 58 % yield with the regio‐chemistry predicted as proposed by transition state **TS‐I** and similar literature strategies.^54^, ^55^, ^56^ The highly reactive ester and Boc‐protected amine provide the scope for a wide range of ring‐closing strategies. In this case, by way of an example, carbamate formation was achieved through ester reduction, using sodium borohydride, followed by base‐mediated intramolecular ring closure. This method furnished spirocycle **3** in moderate yield. The crystal structure of spirocycle **3** conclusively confirmed the regio‐chemical assignment (see SI).

On reviewing the literature, it was hoped that the reaction conditions developed for the acyclic alkene could be used on the exo‐cyclic, electron‐deficient alkenes **5a**–**e**.^39^, ^57^, ^58^, ^59^, ^60^, ^61^ Unfortunately, the reactions of substrates **5a**–**e** failed to proceed under these conditions unlike the substitutions performed on the acyclic, di‐substituted alkenes, and alkenes in strained ring systems reported elsewhere. The lack of progress observed at room temperature was taken to be a consequence of the higher level of steric crowding around the tri‐substituted alkene. We, therefore, looked to explore higher temperatures and pressures. Despite furnishing the desired spirocycles, this provided its own challenges due to the decomposition of the nitrile oxide dipole (**13**) at higher temperatures. Optimisation of these conditions led to the realisation that, similar to Zheng et al., pre‐forming the nitrile oxide dipole (**13**) using triethylamine at 0 °C was required (Scheme 1a).^62^ The generated dipole was then added to the alkene (**5a**–**e**) portion‐wise over 90 minutes under microwave conditions at 100 °C. Unfortunately, despite optimising to these conditions, it was not possible to push the reaction to completion and, as such, the desired spirocycles **7** were all isolated in low to moderate yields ranging between 7 % and 62 % (Table 2).

**Table 2:**
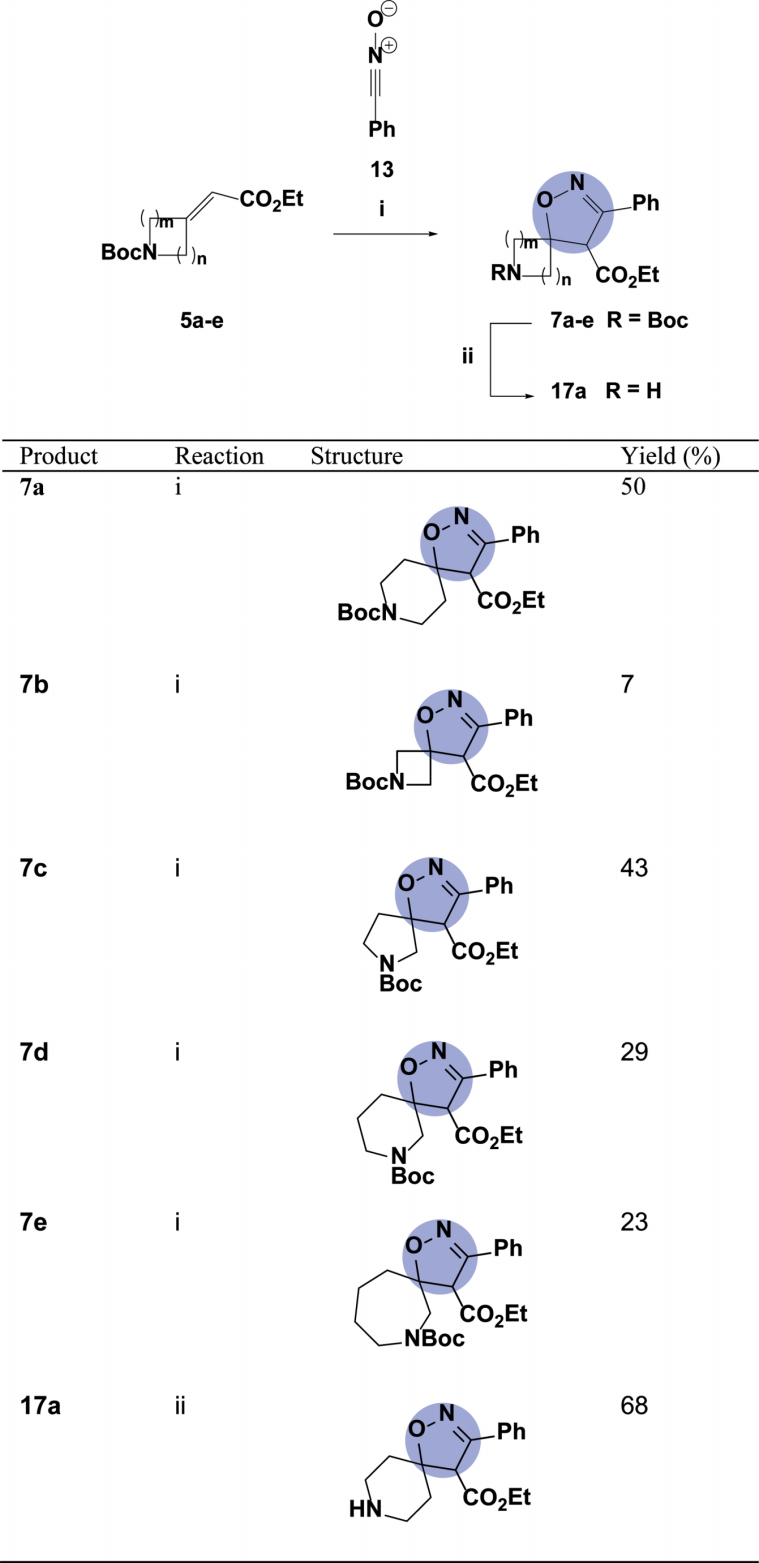
Synthesis of isoxazole‐based spirocycles **7** using freshly‐prepared nitrile‐oxide dipole^a^

a Reaction conditions: (i) **5** (1.0 equiv.), **13** (3 × 2.0 equiv.), DCE, 100 °C (MW), 3 × 30 min; (ii) **7a** (1.0 equiv.), TFA (2.0 equiv.), DCM, r.t., 22 h.

In order for these scaffolds to be used in drug discovery, it would be necessary to remove all protecting groups. This was demonstrated on the 6‐membered spirocycle **7a**, which underwent smooth deprotection using trifluoroacetic acid in dichloromethane to provide secondary amine **17a** in 68 % yield (Table 2).

**Scheme 3:**
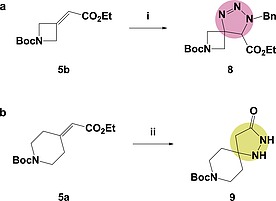
Exploring other alkene cycloadditions: (i) **5b** (1.0 equiv.), BnN_3_ (10.0 equiv.), 100 °C, 48 h, 34 %; (ii) **5a** (1.0 equiv.), NH_2_NH_2_ **·**H_2_O (1.2 equiv.), EtOH, 70 °C, 24 h, 68 %.

Having demonstrated the use of the nitrile oxide dipole for [3+2]‐cycloadditions on both electron‐poor and electron‐rich alkenes we then looked to explore other dipoles. Initially, following a published procedure for similar substrates, a benzyl azide [3+2]‐cycloaddition was attempted involving heating the substrates under reflux.^63^ Again, possibly due to the steric crowding around the alkene, only starting material was isolated. It was only by heating to 100 °C in a sealed vessel for 48 hours that the desired triazole‐based spirocycle **8** was isolated in a moderate 34 % yield (Scheme 3a). Looking beyond [3+2] cycloadditions, hydrazine was used in a one‐pot cyclisation approach (Scheme 3b).

Pleasingly, the Michael‐addition and subsequent substitution formed hydrazide‐based spirocycle **9** in a good 68 % yield.^64^, ^65^ This unusual spirocycle provides multiple handles for further elaboration and therefore would be a valuable addition to a fragment‐screening library.

**Scheme 4:**
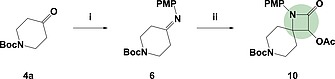
Exploring imine cyclisations: (i) **4a** (1.0 equiv.), *p*‐anisidine (1.0 equiv.), toluene, reflux, 20 h; (ii) **6** (1.0 equiv.), acetoxyacetyl chloride (2.4 equiv.), NEt_3_ (3.0 equiv.), DCM, –78 °C – r.t., 20 h, 55 % over two steps.

Finally, we looked to explore the use of imines in the place of electron‐poor alkenes in a [2+2]‐cycloaddition reaction (Scheme 4). This reaction has good precedent on carbocyclic systems and therefore it was hoped that it would translate cleanly onto a heterocyclic system.^66^ The imine (**6**) was generated from the corresponding ketone **4a** using *para*‐anisidine in toluene. Proving labile under column conditions, **6** was carried through without purification into the [2+2]‐cycloaddition. The imine was trapped with acetoxyacetyl chloride at –78 °C to yield the β‐lactam spirocycle **10** in a moderate 55 % yield over the two steps. Again, multiple handles for further elaboration make this a versatile fragment for FBDD.

Given that the final compounds will be utilised in their de‐protected forms, conditions were identified to remove all protecting groups (Bn, Ac and PMP)^67^, ^68^, ^69^ such that the prophetic structures could be utilised for physicochemical property calculations. A selection of properties was calculated for these final compounds, demonstrating that almost all fragments conformed to the so‐called “Rule of Three”, which provides an important guide to the desirable physicochemical properties of fragment screening libraries.^70^ Of particular note are the low mean value for clogP (0.87) and the high “fraction sp^3^” (0.54), mean number of chiral centres (1.20) and molecular complexity (0.77) (Table 3). Additionally, the molecular shape index (0.55) demonstrates the increased three‐dimensionality of these compounds (where a value of 0 represents spherical and a value of 1 represents linear).

**Table 3:** Mean physicochemical properties

Property^a^	Ideal Range^b^	This Work
**MW**	≤ 300	232.6
**clogP**	0–2	0.87
**PSA**	≤ 60	61.7
**HAC**	10–16	16.8
**HBA**	≤ 3	4.9
**HBD**	≤ 3	1.6
**RBC**	≤ 3	2.6
**Fsp^3^**	–	0.54
**Molecular Shape Index**	–	0.55
**Molecular Complexity**	–	0.77

a MW = molecular weight, PSA = polar surface area, HBA = number of hydrogen‐bond acceptors, HBD = number of hydrogen‐bond donors, HAC = heavy atom count, RBC = rotatable bond count.

b Based on the guidelines used by Astex Pharmaceuticals. See the Supporting Information for further details.

## Conclusions

Spirocycle synthesis remains a challenge within organic chemistry. Heterocyclic spirocycles are particularly synthetically challenging; however, they represent an under‐explored area of chemical space with excellent potential within FBDD. Their restricted conformation reduces the entropic binding penalty and their heterocyclic nature provides opportunities for strong binding interactions as well as potential synthetic handles for elaboration. Overall, we successfully synthesised nine heterocyclic spirocyclic fragments. This collection of scaffolds can be considered substantially diverse since they incorporate [4,5], [4,6], [5,5], [5,6] and [5,7] spirocycles. Furthermore, the types of heterocycles incorporated in the structures vary widely across both rings, including isoxazoles, triazoles, cyclic amines, hydrazides and carbamates. These rings also provide varied types and positions of heteroatoms and thus the potential to identify novel binding interactions. Additionally, we have ensured the presence of functional handles for further elaboration.

We have looked to explore the use of cycloadditions as a synthetic strategy for heterocyclic spirocycle formation as they enable regio‐selective and short divergent syntheses. Despite the challenges encountered conducting these types of reactions on already‐sterically‐hindered centres, the synthesis of nine diverse, novel spirocycles has introduced the powerful potential of this synthetic strategy and highlights the need for further work in this area.

## Experimental Section

All reactions were carried out under nitrogen atmosphere unless otherwise stated. Solvents and commercially available reagents were dried and purified before use, where appropriate using standard procedures. Toluene, hexane, diethyl ether, ethyl acetate (EtOAc), petroleum ether (fraction 40–60), methanol, THF, and dichloromethane (DCM) were dried and distilled using standard methods from oxygen free from solvent dispenser units under an argon atmosphere. Analytical thin layer chromatography (TLC) was performed using pre‐coated Merck glass‐backed silica gel plates (Silica gel 60 F254). Flash column chromatography was undertaken on Fluka or Material Harvest silica gel (230–400 mesh).

Proton and carbon magnetic resonance spectra were recorded using an internal deuterium lock (at 298 K unless stated otherwise) on Bruker DPX (400/101 MHz; ^1^H‐^13^C DUL probe), Bruker Avance III HD (400/101 MHz; Smart probe), Bruker Avance III HD (500/126 MHz; Smart probe) and Bruker Avance III HD (500/126 MHz; DCH Cryoprobe) spectrometers. Chemical shifts (*δ*_H_) are quoted in ppm to the nearest 0.01 ppm and are referenced to the residual non‐deuterated solvent peak. Data are reported as: chemical shift, multiplicity (br = broad; s = singlet; d = doublet; t = triplet; q = quartet; qn = quintet; sp = septet; m = multiplet; or a combination thereof), coupling constants, number of nuclei, and assignment.

Infrared (IR) spectra were recorded on a Perkin Elmer 1FT‐IR Spectrometer fitted with an ATR sampling accessory as either solids or neat films, either through direct application or deposited in CDCl_3_. Absorption maxima (*ν*_max_) are reported in wavenumbers (cm^–1^) with the following abbreviations: w, weak; m, medium; s, strong; br, broad. High‐resolution mass spectra (HRMS) were measured on a Micromass LCT Premier spectrometer using electron spray ionization (ESI) techniques. Masses are quoted within the 5 ppm error limit. Melting points were obtained on a Buchi B‐545 melting point apparatus and are uncorrected. Chiral products had their optical rotation recorded on an Anton‐Paar MCP 100 polarimeter. [*α*]_D_^20^ values are reported in ° g^–1^ cm^–2^ × 10^–1^ at the sodium D‐line of 598 nm, concentration (*c*) is given in g(100 mL)^–1^ in the solvent stated.

***N*‐Hydroxybenzimidoyl Chloride (12):** Benzaldehyde **11** (1.5 g, 14.1 mmol, 1.0 equiv.) was dissolved in diethyl ether (12 mL) and hydroxylamine (50 % w/w in H_2_O, 1.73 mL, 29.3 mmol, 2.0 equiv.) was added. The reaction was stirred for 10 minutes, dried (MgSO_4_), filtered and concentrated in vacuo. The residual oil was redissolved in *N*,*N*‐dimethylformamide (12 mL), *N*‐chlorosuccinimide (2.1 g, 15.6 mmol, 1.1 equiv.) was added and the reaction was stirred for 30 minutes. The reaction was diluted with water (10 mL) and extracted with ethyl acetate (2 × 10 mL). The combined organic extracts were washed with water (20 mL) and brine (20 mL), dried (Na_2_SO_4_), filtered and concentrated in vacuo to give the crude product as a yellow oil, which was used directly without further purification (2.2 g, 14.0 mmol, 99 %); *R*_f_: 0.45 (Pet. Ether 40–60/EtOAc, 4:1); IR (thin film) ν̃ /cm^–1^: 3246 (O‐H, m), 3058 (Ar‐H, m), 1650 (C=N, s); ^1^H NMR (400 MHz, CDCl_3_, 25 °C) *δ* /ppm: 8.94 (br s, 1H), 7.89–7.86 (m, 2H), 7.47–7.41 (m, 3H); HRMS (ESI) C_7_H_7_NOCl *m/z*: [M + H]^+^ 156.0220 (calc. 156.0126). Data agree with that reported in the literature.^48^

**Ethyl (*tert*‐Butoxycarbonyl)‐L‐serinate (15):** L‐serine ethyl ester hydrochloride **14** (5 g, 29.48 mmol, 1.0 equiv.) and di‐*tert*‐butyl dicarbonate (6.43 mL, 28.01 mmol, 0.95 equiv.) were dissolved in ethyl acetate (40 mL) and water (40 mL). Potassium carbonate (6.11 g, 44.22 mmol, 1.5 equiv.) was added to the reaction and it was left to stir overnight at r.t. The layers were then separated and the aqueous extracted with ethyl acetate (2 × 30 mL) and the combined organics were washed with brine (50 mL). This was then dried with anh. MgSO_4_. This mixture was filtered and the filtrate was concentrated in vacuo to give ethyl (*tert*‐butoxycarbonyl)‐L‐serinate **15** as a pure colourless oil (6.41 g, 27.49 mmol, 93 %); *R*_f_: 0.07 (Pet. Ether 40–60/EtOAc, 4:1); IR (thin film) ν̃/cm^–1^: 3383 (O‐H, br s), 2978 (C‐H, w), 1714 (C=O ester, s), 1692 (C=O carbamide, s), 1158 (C‐O, s); ^1^H NMR (400 MHz, CDCl_3_) *δ*/ppm: 5.44 (br s, 1H), 4.36 (br s, 1H), 4.24 (q, *J* = 7.0 Hz, 2H), 3.98–3.89 (m, 2H), 2.30 (br s, 1H), 1.46 (s, 9H), 1.30 (t, *J* = 7.0 Hz, 3H); ^13^C NMR (101 MHz, CDCl_3_) *δ*/ppm: 170.7, 155.8, 80.3, 63.8, 61.8, 55.8, 28.3, 14.1; HRMS (ESI) C_10_H_19_NO_5_Na *m/z*: [M + Na]^+^ 256.1151 (*calc*. 256.1155). [*α*]_D_^20^ = –17.2 (*c* = 1.0, CHCl_3_). Data agree with that reported in the literature.^71^

**Ethyl 2‐[(*tert*‐Butoxycarbonyl)amino]acrylate (1):** Ethyl (*tert*‐butoxycarbonyl)‐L‐serinate **15** (6.4 g, 27.44 mmol, 1.0 equiv.) was dissolved in dichloromethane (70 mL) at –15 °C. Mesyl chloride (2.65 mL, 34.30 mmol, 1.25 equiv.) was added dropwise followed by triethylamine (11.41 mL, 82.31 mmol, 3.0 equiv.). The reaction was stirred for 30 min before being warmed to r.t. and stirred for a further 2 h. Then one third molar HCl (30 mL) was added. The mixture was washed with water and the combined organics were dried with anh. MgSO_4_. This mixture was filtered, and the filtrate was concentrated in vacuo to give the crude product. This was filtered through Celite with dichloromethane to afford ethyl 2‐[(*tert*‐butoxycarbonyl)amino]acrylate **1** as a yellow oil (5.5667 g, 25.86 mmol, 94 %); *R*_f_: 0.60 (Pet. Ether 40–60/EtOAc, 4:1); IR (thin film) ν̃/cm^–1^: 3420 (N‐H, w), 2980 (C‐H, w), 1733 (C=O carbamate, m), 1707 (C=O ester, s), 1638 (C=C, w), 1152 (C‐O, s); ^1^H NMR (400 MHz, CDCl_3_) *δ*/ppm: 7.02 (br s, 1H), 6.14 (br s, 1H), 5.72 (d, *J* = 1.5 Hz, 1H), 4.28 (q, *J* = 7.0 Hz, 2H), 1.48 (s, 9H), 1.33 (t, *J* = 7.0 Hz, 3H); ^13^C NMR (101 MHz, CDCl_3_) *δ*/ppm: 164.0, 152.6, 131.5, 104.8, 80.6, 62.0, 28.2, 14.1; HRMS (ESI) C_10_H_17_NO_4_Na *m/z*: [M + Na]^+^ 238.1046 (*calc*. 238.1050). Data agree with that reported in the literature.^49^

**General Procedure for Synthesis of α,β‐Unsaturated Esters (5):** *N*‐Boc heterocyclic ketone **4** (1 equiv.) and ethyl (triphenylphosphoranylidine) acetate **16** (1.1 equiv.) were dissolved in dichloromethane and refluxed. Upon complete conversion of starting material, the mixtures were concentrated in vacuo and submitted to flash column chromatography to yield the pure products.

***tert*‐Butyl 4‐(2‐Ethoxy‐2‐oxoethylidene)piperidine‐1‐carboxylate (5a):** *tert*‐Butyl 4‐oxopiperidine‐1‐carboxylate **4a** (3.50 g, 17.6 mmol, 1.0 equiv.) and ethyl (triphenylphosphoranylidene)acetate **16** (6.73 g, 19.3 mmol, 1.1 equiv.) were dissolved in dichloromethane (50 mL) and refluxed for 17 hours. The reaction was concentrated in vacuo and submitted to column chromatography (Pet. Ether 40–60/EtOAc, 9:1 to 4:1) to yield the product, *tert*‐butyl 4‐(2‐ethoxy‐2‐oxoethylidene) piperidine‐1‐carboxylate **5a** as a colourless, crystalline solid (2.53 g, 9.4 mmol, 53 %); *R*_f_: 0.24 (Pet. Ether 40–60/EtOAc, 4:1); IR (thin film) ν̃ /cm^–1^: 2972 (C‐H, w), 1706 (C=O ester, s), 1676 (C=O carbamate, s), 1143 (C‐O, s); ^1^H NMR (400 MHz, CDCl_3_, 25 °C) *δ* /ppm: 5.71 (m, 1H), 4.15 (q, *J* = 7.2 Hz, 2H), 3.49 (app. quin, *J* = 5.7 Hz, 4H), 2.93 (t, *J* = 5.5 Hz, 2H), 2.27 (t, *J* = 5.6 Hz, 2H) 1.47 (s, 9H), 1.28 (t, *J* = 7.1, 3H); ^13^C NMR (100 MHz, CDCl_3_) *δ* /ppm: 166.5, 158.0, 154.7, 115.4, 80.0, 59.9, 36.6, 29.7, 28.5, 14.4; m.p. 84–86 °C (Lit. Value 84–86 °C); HRMS (ESI) C_14_H_23_NO_4_Na *m/z*: [M + Na]^+^ 292.1515 (calc. 292.1519). Data agree with that reported in the literature.^72^, ^73^

***tert*‐Butyl 3‐(2‐Ethoxy‐2‐oxoethylidene)azetidine‐1‐carboxylate (5b):** *tert*‐Butyl 3‐oxoazetidine‐1‐carboxylate **4b** (2.90 g, 16.9 mmol, 1.0 equiv.) and ethyl (triphenylphosphoranylidene)acetate **16** (6.49 g, 18.6 mmol, 1.1 equiv.) were dissolved in dichloromethane (56 mL) and refluxed for 6 h. The reaction was concentrated in vacuo and submitted to column chromatography (Pet. Ether 40–60/EtOAc, 4:1) to yield the pure product, *tert*‐butyl 3‐(2‐ethoxy‐2‐oxoethylidene)azetidine‐1‐carboxylate **5b** as a colourless oil (3.85 g, 16.0 mmol, 94 %); *R*_f_: 0.29 (Pet. Ether 40–60/EtOAc, 4:1); IR (thin film) ν̃/cm^–1^: 2978 (C‐H, w), 1725 (C=O carbamate, s), 1704 (C=O ester, s), 1197 (C‐O, s); ^1^H NMR (400 MHz, CDCl_3_, 25 °C) *δ* /ppm: 5.76 (quin, *J* = 2.2 Hz, 1H), 4.81 (m, 2H), 4.58 (m, 2H), 4.17 (q, *J* = 7.1 Hz, 2H), 1.45 (s, 9H), 1.27 (t, *J* = 7.2 Hz, 3H); ^13^C NMR (100 MHz, CDCl_3_) *δ* /ppm: 165.2, 156.2, 152.6, 113.7, 80.1, 60.4, 57.8, 28.3, 14.3; HRMS (ESI) C_12_H_19_NO_4_Na *m/z*: [M + Na]^+^ 264.1215 (calc. 264.1212). Data agree with that reported in the literature.^41^

***tert*‐Butyl 3‐(2‐Ethoxy‐2‐oxoethylidene)pyrrolidine‐1‐carboxylate (5c):** *tert*‐Butyl 3‐oxopyrrolidine‐1‐carboxylate **4c** (0.50 g, 2.69 mmol, 1.0 equiv.) and ethyl (triphenylphosphoranylidene)acetate **16** (1.03 g, 2.97 mmol, 1.1 equiv.) were dissolved in dichloromethane (9 mL) and refluxed for 20 h. The reaction was concentrated in vacuo and submitted to column chromatography (Pet. Ether 40–60/EtOAc, 9:1 to 3:1) to yield the *E/Z* isomers of the product *tert*‐butyl 3‐(2‐ethoxy‐2‐oxoethylidene) pyrrolidine‐1‐carboxylate **5c** as colourless oils. (0.45 g, 1.76 mmol, combined *E/Z* yield 65 %); *R*_f_: 0.23 (*E*) and 0.18 (*Z*) (Pet. Ether 40–60/EtOAc, 4:1); (*E*)‐isomer: IR (thin film) ν̃ /cm^–1^: 2979 (C‐H, w), 1710 (C=O ester, s), 1670 (C=O carbamate, s), 1212 (C‐O, s), 1165 (C‐O, s); ^1^H NMR (400 MHz, CDCl_3_, 25 °C) *δ* /ppm: 5.82 (br s, 1H), 4.16 (q, *J* = 7.1 Hz, 2H), 4.11 (br s, 2H), 3.56 (br s, 2H), 3.11 (br s, 2H), 1.46 (s, 9H), 1.27 (t, *J* = 7.1 Hz, 3H); ^13^C NMR (100 MHz, CDCl_3_) *δ* /ppm: 166.1, 154.4, 122.4, 112.8, 83.8, 80.0, 77.4, 60.5, 45.7, 28.6, 14.4; (*Z*)‐isomer: IR (thin film) ν̃ /cm^–1^: 2978 (C‐H, w), 1702 (C=O ester, s), 1676 (C=O carbamate, s), 1215 (C‐O, s), 1163 (C‐O, s); ^1^H NMR (400 MHz, CDCl_3_, 25 °C) *δ* /ppm: 5.80 (m, 1H), 4.41 (s, 2H), 4.16 (q, *J* = 7.1 Hz, 2H), 3.50 (m, 2H), 2.73 (m, 2H), 1.46 (s, 9H), 1.27 (t, *J* = 7.1 Hz, 3H); ^13^C NMR (100 MHz, CDCl_3_) *δ* /ppm: 166.1, 160.6, 154.5, 113.0, 79.8, 60.2, 50.6, 43.8, 32.9, 28.6, 14.4; HRMS (ESI) C_13_H_21_NO_4_Na *m/z*: [M + Na]^+^ 278.1356 (calc. 278.1363). Data agreed with that reported in the literature.^74^

***tert*‐Butyl 3‐(2‐Ethoxy‐2‐oxoethylidene)piperidine‐1‐carboxylate (5d):** *tert*‐Butyl 3‐oxopiperidine‐1‐carboxylate **4d** (4.00 g, 20.1 mmol, 1.0 equiv.) and ethyl (triphenylphosphoranylidene)acetate **16** (7.69 g, 22.1 mmol, 1.1 equiv.) were dissolved in dichloromethane (50 mL) and refluxed for 18 hours. The reaction was concentrated in vacuo and submitted to column chromatography (Pet. Ether 40–60/EtOAc, 9:1 to 3:1) to yield the *E/Z* isomers of the product, *tert*‐butyl 3‐(2‐ethoxy‐2‐oxoethylidene) piperidine‐1‐carboxylate **5d** as colourless oils (2.55 g, 9.5 mmol, combined *E/Z* yield 47 %); *R*_f_: 0.32 (*E*) and 0.25 (*Z*) (Pet. Ether 40–60/EtOAc, 4:1); (*E*)‐isomer: IR (thin film) ν̃ /cm^–1^: 2978 (C‐H, w), 1692 (C=O ester, s), 1659 (C=O carbamate, s), 1150 (C‐O, s); ^1^H NMR (400 MHz, CDCl_3_, 25 °C) *δ* /ppm: 5.73 (br s, 1H), 4.14 (q, *J* = 7.1 Hz, 2H), 3.92 (br s, 2H), 3.47 (m, 2H), 2.92 (m, 2H), 1.68 (m, 2H), 1.44 (s, 9H), 1.26 (t, *J* = 7.1 Hz, 3H); ^13^C NMR (100 MHz, CDCl_3_) *δ* /ppm: 166.5, 154.7, 115.7, 80.0, 60.0, 51.4, 44.5, 28.5, 27.7, 25.4, 14.4; (*Z*)‐isomer: IR (thin film) ν̃ /cm^–1^: 2978 (C‐H, w), 1692 (C=O ester, s), 1662 (C=O carbamate, s), 1212 (C‐O, s), 1154 (C‐O, s); ^1^H NMR (400 MHz, CDCl_3_, 25 °C) *δ* /ppm: 5.65 (br s, 1H), 4.60 (s, 2H), 4.16 (q, *J* = 7.1 Hz, 2H), 3.47 (m, 2H), 2.33 (m, 2H), 1.71 (m, 2H), 1.44 (s, 9H), 1.27 (t, *J* = 7.1 Hz, 3H); ^13^C NMR (100 MHz, CDCl_3_) *δ* /ppm: 165.9, 155.0, 154.6, 115.5, 79.9, 60.1, 43.4, 34.2, 28.5, 27.6, 25.6, 14.4; HRMS (ESI) C_14_H_23_NO_4_Na *m/z*: [M + Na]^+^ 292.1521 (calc. 292.1519). Data agree with that reported in the literature.^75^

***tert*‐Butyl 3‐(2‐Ethoxy‐2‐oxoethylidene)azepane‐1‐carboxylate (5e):** *tert*‐Butyl 3‐oxoazepane‐1‐carboxylate **4e** (0.50 g, 2.34 mmol, 1.0 equiv.) and ethyl (triphenylphosphoranylidene)acetate **16** (4.08 g, 11.7 mmol, 5.0 equiv.) were dissolved in toluene (20 mL) and refluxed for 44 hours. The reaction was concentrated in vacuo and submitted to column chromatography (Pet. Ether 40–60/EtOAc, 19:1 to 4:1) to yield an isomeric mixture of the products, (*E*)‐ and (*Z*)‐*tert*‐Butyl 3‐(2‐ethoxy‐2‐oxoethylidene)azepane‐1‐carboxylate **5e** as an orange oil (0.52 g, 1.85 mmol, 80 %); *R*_f_: 0.51 (Pet. Ether 40–60/EtOAc, 7:3); IR (thin film) ν̃ /cm^–1^: 2936 (C‐H, w), 1689 (C=O ester, s), 1645 (C=O carbamate, s), 1152 (C‐O, s); ^1^H NMR (400 MHz, CDCl_3_, 25 °C) *δ* /ppm: 5.73 & 5.70 (s, 1H), 4.16 & 4.15 (q, *J* = 7.1 Hz, 2H), 4.12 & 4.04 (s, 2H), 3.29 & 3.20 (m, 2H), 2.79 & 2.74 (m, 2H), 1.75–1.74 (m, 4H), 1.47 & 1.45 (s, 9H), 1.28 & 1.27 (t, *J* = 7.1 Hz, 3H); ^13^C NMR (100 MHz, CDCl_3_) *δ* /ppm: 166.2, 166.1, 161.4, 160.8, 155.7, 155.1, 116.7, 116.3, 80.0, 79.8, 59.9, 59.8, 55.5, 54.7, 47.4, 47.2, 30.2, 29.7, 29.0, 28.9, 28.6, 28.5, 27.8, 27.3, 14.4, 14.4; HRMS (ESI) C_15_H_25_NO_4_Na *m/z*: [M + Na]^+^ 306.1678 (calc. 306.1676). Data agree with that reported in the literature.^75^

**Ethyl 4‐[(*tert*‐Butoxycarbonyl)amino]‐3‐phenyl‐4,5‐dihydroisoxazole‐4‐carboxylate (2):** Triethylamine (2.2 mL, 15.80 mmol, 6.8 equiv.) was added to a mixture of ethyl 2‐[(*tert*‐butoxycarbonyl)amino]acrylate **1** (500 mg, 2.32 mmol, 1.0 equiv.) and *N*‐hydroxybenzimidoyl chloride **12** (2.17 g, 13.94 mmol, 6.0 equiv.) in dichloromethane (40 mL) and the reaction was stirred at r.t. for 1 h. Water (30 mL) was added and the organic layer was washed with water (30 mL) and brine (30 mL). The combined organics were dried with anh. MgSO_4_. This mixture was filtered, and the filtrate was concentrated in vacuo to give the crude product. This was purified by column chromatography (Pet. Ether 40–60/Et_2_O, 4:1) to give ethyl 4‐[(*tert*‐butoxycarbonyl)amino]‐3‐phenyl‐4,5‐dihydroisoxazole‐4‐carboxylate **2**, as off‐white amorphous solid (448.6 mg, 1.34 mmol, 58 %); *R*_f_: 0.12 (20 % diethyl ether in pet. ether); IR (thin film) ν̃ /cm^–1^: 3410 (N‐H, m), 2976 (C‐H, w), 1741 (C=O, m), 1721 (C=N, s), 1484 (C=C, s), 1308 (N‐O, s); ^1^H NMR (400 MHz, CDCl_3_) *δ*/ppm: 7.70–7.66 (m, 2H, H11), 7.43–7.38 (m, 3H, H12&13), 6.08 (br s, 1H, NH), 4.32 (q, *J* = 7.0 Hz, 2H), 3.91 (br s, 2H), 1.40 (s, 9H), 1.32 (t, *J* = 7.0 Hz, 3H); ^13^C NMR (101 MHz, CDCl_3_) *δ*/ppm: 167.6, 156.3, 153.3, 130.4, 129.8, 128.7, 126.9, 92.4, 81.2, 63.4, 42.7, 28.2, 14.0; HRMS (ESI) C_17_H_23_N_2_O_5_ *m/z*: [M + H]^+^ 335.1591 (calc. 335.1607).

***tert*‐Butyl [4‐(Hydroxymethyl)‐3‐phenyl‐4,5‐dihydroisoxazol‐4‐yl]carbamate (2′):** Ethyl 4‐[(*tert*‐butoxycarbonyl)amino]‐3‐phenyl‐4,5‐dihydroisoxazole‐4‐carboxylate **2** (100 mg, 0.30 mmol, 1.0 equiv.), was dissolved in an 8:1 THF/methanol mixture (1.8 mL). This was cooled to 0 °C and sodium borohydride (2.83 mg, 0.07 mmol, 0.25 equiv.) were added. The reaction was stirred for 4 h before further sodium borohydride (8.49 mmol, 0.21 mmol, 0.75 equiv.) was added. The reaction was stirred overnight. It was quenched with sat. aq. NH_4_Cl (2 mL), and extracted with dichloromethane (3 × 5 mL). The combined organics were then dried with anh. MgSO_4_. This mixture was filtered, and the filtrate was concentrated in vacuo to give the crude product. This was purified via column chromatography (hexane/EtOAc, 1:1) to give *tert*‐butyl [4‐(hydroxymethyl)‐3‐phenyl‐4,5‐dihydroisoxazol‐4‐yl]carbamate **2′** as a white powder (54.0 mg, 0.18 mmol, 62 %); *R*_f_: 0.29 (55 % ethyl acetate in hexane); IR (thin film) ν̃/cm^–1^: 3408 (N‐H, w), 3278 (O‐H, w), 2970 (C‐H, w), 1717 (C=O, s), 1545 (C=C, s); ^1^H NMR (400 MHz, CDCl_3_) *δ*/ppm: 7.66–7.64 (m, 2H), 7.40–7.37 (m, 3H), 5.74 (br s, 1H), 3.90–3.86 (m, 2H), 3.77–3.73 (m, 1H), 3.30 (d, *J* = 17.5 Hz, 1H), 3.05 (br s, 1H), 1.40 (s, 9H); ^13^C NMR (101 MHz, CDCl_3_) *δ*/ppm: 157.3, 154.1, 130.3, 129.6, 128.7, 126.7, 95.4, 80.9, 66.2, 40.4, 28.2; HRMS (ESI) C_15_H_21_N_2_O_4_ *m/z*: [M + H]^+^ 293.1498 (*calc*. 293.1501).

**3‐Phenyl‐1,8‐Dioxa‐2,6‐diazaspiro[4.4]non‐2‐en‐7‐one (3):** To a solution of potassium *tert*‐butoxide (30.7 mg, 0.27 mmol, 2.0 equiv.) in THF (1.4 mL) at 0 °C was added *tert*‐butyl [4‐(hydroxymethyl)‐3‐phenyl‐4,5‐dihydroisoxazol‐4‐yl]carbamate **2′** (40 mg, 0.14 mmol, 1.0 equiv.) and the reaction was stirred for 1 h. This was then diluted with sat. aq. NH_4_Cl (5 mL) and extracted with dichloromethane (3 × 5 mL). The combined organics were dried with anh. MgSO_4_, filtered and concentrated in vacuo to give the crude product. This was purified by column chromatography (60 % ethyl acetate in hexane) to give 3‐phenyl‐1,8‐dioxa‐2,6‐diazaspiro[4.4]non‐2‐en‐7‐one **3** as a white powder (17 mg, 0.08 mmol, 56 %); *R*_f_: 0.16 (hexane/EtOAc, 2:3); IR (thin film) ν̃ /cm^–1^: 3226 (N‐H, w), 1765 (C=O, s), 1048 (C‐O, s); ^1^H NMR (400 MHz, CDCl_3_) *δ*/ppm: 7.64–7.62 (m, 2H), 7.48–7.41 (m, 3H), 5.97 (br s, 1H), 4.77 (d, *J* = 10.0 Hz, 1H), 4.59 (d, *J* = 10.0 Hz, 1H), 3.59 (d, *J* = 18.0 Hz, 1H), 3.49 (d, *J* = 18.0 Hz, 1H); ^13^C NMR (101 MHz, CDCl_3_) *δ*/ppm: 156.3, 156.2, 131.1, 129.1, 128.2, 126.6, 96.1, 73.9, 42.8; HRMS (ESI) C_11_H_10_N_2_O_3_Na *m/z*: [M + Na]^+^ 241.0576 (calc 241.0584). M.p. 178–179 °C. For crystallographic data, see SI.

**General Method for the Cycloaddition of α,β‐Unsaturated Esters with *N*‐Hydroxybenzimidoyl Chloride (7):** To a stirred solution of *N*‐hydroxybenzimidoyl chloride **12** (6 equiv.) in dichloroethane (2 mL) at 0 °C was added triethylamine (6 equiv.) dropwise. The resulting suspension was stirred for 5 minutes and then washed with water (2 × 2 mL), dried (MgSO_4_) and filtered to leave a solution of nitrile oxide intermediate **13** in dichloroethane at 0 °C. A solution of α,β‐unsaturated ester **3** (1 equiv.) was prepared in dichloroethane (2 mL). To this solution was added approximately 1 equivalent of the nitrile oxide solution and the mixture was heated at 100 °C (MW) for 30 minutes. Further additions and resubmission to heating conditions were performed if the reaction looked incomplete. The mixture was then concentrated and submitted to flash column chromatography to yield the pure products.

**8‐(*tert*‐Butyl) 4‐Ethyl 3‐Phenyl‐1‐oxa‐2,8‐diazaspiro[4.5]dec‐2‐ene‐4,8‐dicarboxylate (7a):** Following the general procedure for *N*‐hydroxybenzimidoyl chloride cycloaddition, *tert*‐butyl 4‐(2‐ethoxy‐2‐oxoethylidene)piperidine‐1‐carboxylate **5a** (50 mg, 0.19 mmol) furnished the product, 8‐(*tert*‐butyl) 4‐ethyl 3‐phenyl‐1‐oxa‐2,8‐diazaspiro[4.5]dec‐2‐ene4,8‐dicarboxylate **7a**, as a colourless oil after 3 consecutive submissions (36 mg, 0.09 mmol, 50 %); *R*_f_: 0.18 (Pet. Ether 40–60/EtOAc, 4:1); IR (thin film) ν̃ /cm^–1^: 2978 (C‐H, m), 1732 (C=O ester, s), 1687 (C=O carbamate/C=N, s), 1139 (C‐O, s); ^1^H NMR (400 MHz, CDCl_3_, 25 °C) *δ* /ppm: 7.61 (m, 2H), 7.39 (m, 3H), 4.17 (q, *J* = 7.1 Hz, 2H), 4.10 (s, 1H), 3.95 (m, 2H), 3.28 (m, 2H), 1.98–1.61 (m, 4H), 1.47 (s, 9H), 1.19 (t, *J* = 7.1 Hz, 3H); ^13^C NMR (100 MHz, CDCl_3_) *δ* /ppm: 167.7, 155.2, 154.7, 130.4, 129.2, 129.0, 126.5, 87.0, 80.0, 62.3, 62.0, 36.8, 31.2, 28.6, 14.2; HRMS (ESI) C_21_H_28_N_2_O_5_Na *m/z*: [M + Na]^+^ 411.1888 (calc. 411.1890).

**2‐(*tert*‐Butyl) 8‐Ethyl 7‐Phenyl‐5‐oxa‐2,6‐diazaspiro[3.4]oct‐6‐ene‐2,8‐dicarboxylate (7b):** Following the general procedure for *N*‐hydroxybenzimidoyl chloride cycloaddition, *tert*‐butyl 3‐(2‐ethoxy‐2‐oxoethylidene)azetidine‐1‐carboxylate **5b** (50 mg, 0.21 mmol) furnished the product, 2‐(*tert*‐butyl) 8‐ethyl 7‐phenyl‐5‐oxa‐2,6‐diazaspiro[3.4]oct‐6‐ene2,8‐dicarboxylate **7b**, as a colourless oil after 3 consecutive submissions (5.4 mg, 0.01 mmol, 7 %); *R*_f_: 0.31 (Pet. Ether 40–60/EtOAc, 2:1); IR (thin film) ν̃ /cm^–1^: 2978 (C‐H, w), 1736 (C=O ester, s), 1698 (C=O carbamate/C=N, s), 1392 (N‐O, s), 1153 (C‐O, s); ^1^H NMR (400 MHz, CDCl_3_, 25 °C) *δ* /ppm: 7.68 (m, 2H), 7.42 (m, 3H), 4.45 (s, 1H), 4.34 (dd, *J* = 10.8 Hz, 0.8 Hz, 1H), 4.27–4.15 (m, 4H), 4.05 (dd, *J* = 9.8 Hz, 0.9 Hz, 1H), 1.46 (s, 9H), 1.20 (t, *J* = 7.1 Hz, 3H); ^13^C NMR (100 MHz, CDCl_3_) *δ* /ppm: 167.0, 155.9, 154.8, 130.8, 128.9, 128.0, 126.8, 83.4, 80.3, 64.7, 62.4, 60.5, 57.3, 28.3, 14.0; HRMS (ESI) C_19_H_25_N_2_O_5_ *m/z*: [M + H]^+^ 361.6087 (calc. 361.6085).

**7‐(*tert*‐Butyl) 4‐Ethyl 3‐Phenyl‐1‐oxa‐2,7‐diazaspiro[4.4]non‐2‐ene‐4,7‐dicarboxylate (7c):** Following the general procedure for *N*‐hydroxybenzimidoyl chloride cycloaddition, *tert*‐butyl 3‐(2‐ethoxy‐2‐oxoethylidene)pyrrolidine‐1‐carboxylate **5c** (50 mg, 0.20 mmol) furnished the product, 7‐(*tert*‐butyl) 4‐ethyl 3‐phenyl‐1‐oxa‐2,7‐diazaspiro[4.4]non‐2‐ene4,7‐dicarboxylate **7c**, as a colourless oil after 3 consecutive submissions (24 mg, 0.09 mmol, 43 %); *R*_f_: 0.24 (Pet. Ether 40–60/EtOAc, 7:3); IR (thin film) ν̃ /cm^–1^: 3375 (Ar‐H, w), 2980 (C‐H, m), 1728 (C=O ester, s), 1704 (C=O carbamate/C=N, s), 1148 (C‐O, s); ^1^H NMR (400 MHz, CDCl_3_, 25 °C) *δ* /ppm: 7.66 (m, 2H), 7.41 (m, 3H), 4.83 (s, 1H), 4.19 (m, 2H), 3.86 (m, 2H), 2.45 (m, 1H), 2.19 (m, 1H), 1.55 (s, 9H), 1.19 (t, *J* = 7.5 Hz, 3H); ^13^C NMR (100 MHz, CDCl_3_) *δ* /ppm: 169.0, 167.6, 155.3, 150.0, 130.8, 129.0, 127.1, 90.8, 84.2, 62.4, 57.2, 43.5, 28.6, 28.1, 27.0, 14.2; HRMS (ESI) C_20_H_26_N_2_O_5_Na *m/z*: [M + Na]^+^ 397.1738 (calc. 397.1734).

**7‐(*tert*‐Butyl) 4‐Ethyl 3‐Phenyl‐1‐oxa‐2,7‐diazaspiro[4.5]dec‐2‐ene‐4,7‐dicarboxylate (7d):** Following the general procedure for *N*‐hydroxybenzimidoyl chloride cycloaddition, *tert*‐butyl 3‐(2‐ethoxy‐2‐oxoethylidene)piperidine‐1‐carboxylate **5d** (50 mg, 0.19 mmol) furnished the product, 7‐(*tert*‐butyl) 4‐ethyl 3‐phenyl‐1‐oxa‐2,7‐diazaspiro[4.5]dec‐2‐ene4,7‐dicarboxylate **7d**, as a colourless oil after 3 consecutive submissions (21 mg, 0.06 mmol, 29 %); *R*_f_: 0.29 (Pet. Ether 40–60/EtOAc, 2:1); IR (thin film) ν̃ /cm^–1^: 2975 (C‐H, m), 1732 (C=O ester, s), 1687 (C=O carbamate/C=N, s), 1150 (C‐O, s); ^1^H NMR (400 MHz, CDCl_3_, 25 °C) *δ* /ppm: 7.64 (m, 2H), 7.40 (m, 3H), 4.17 (q, *J* = 7.1 Hz, 2H), 4.05 (m, 1H), 3.59 (m, 2H), 3.32 (m, 2H), 1.99 (m, 2H), 1.83 (m, 1H), 1.66 (m, 1H), 1.45 (br s, 9H), 1.18 (t, *J* = 7.12, 3H); ^13^C NMR (100 MHz, CDCl_3_) *δ* /ppm: 167.8, 155.4, 154.8, 130.5, 129.1, 129.0, 126.6, 86.9, 80.3, 62.0, 61.2, 58.8, 43.2, 29.9, 28.4, 22.8, 14.1; HRMS (ESI) C_21_H_28_N_2_O_5_Na *m/z*: [M + Na]^+^ 411.1901 (calc. 411.1890).

**7‐(*tert*‐Butyl) 4‐Ethyl 3‐Phenyl‐1‐oxa‐2,7‐diazaspiro[4.6]undec‐2‐ene‐4,7‐dicarboxylate (7e):** Following the general procedure for *N*‐hydroxybenzimidoyl chloride cycloaddition, *tert*‐butyl 3‐(2‐ethoxy‐2‐oxoethylidene)azepane‐1‐carboxylate **5e** (50 mg, 0.18 mmol) furnished the product, 7‐(*tert*‐butyl) 4‐ethyl 3‐phenyl‐1‐oxa‐2,7‐diazaspiro[4.6]undec‐2‐ene‐4,7‐dicarboxylate **7e**, as a colourless oil after 3 consecutive submissions (17 mg, 0.04 mmol, 23 %); *R*_f_: 0.33 (Pet. Ether 40–60/EtOAc, 2:1); IR (thin film) ν̃ /cm^–1^: 2931 (C‐H, m), 1722 (C=O ester, s), 1682 (C=O carbamate/C=N, s), 1158 (C‐O, s); ^1^H NMR (400 MHz, CDCl_3_, 25 °C) *δ* /ppm: 7.64 (m, 2H), 7.38 (m, 3H), 4.80 & 4.38 (s, ′1H′), 4.16 (m, 2H), 3.77 (m, 1H), 3.76 & 3.39–3.02 (m, 2H), 1.99 (m, 1H), 1.80 (m, 3H), 1.62 (m, 2H), 1.49 & 1.31 (s, ′9H′), 1.18 (m, 3H); ^13^C NMR (100 MHz, CDCl_3_) *δ* /ppm: 168.2, 155.7, 155.5, 130.4, 129.0, 127.0, 126.6, 92.4, 80.7, 61.9, 59.3, 53.4, 48.7, 34.4, 28.5, 28.3, 22.3, 14.2; HRMS (ESI) C_22_H_30_N_2_O_5_Na *m/z*: [M + Na]^+^ 425.2048 (calc. 425.2047).

**Ethyl 3‐Phenyl‐1‐oxa‐2,8‐diazaspiro[4.5]dec‐2‐ene‐4‐carboxylate (17a):** To a solution of 8‐(*tert*‐butyl) 4‐ethyl 3‐phenyl‐1‐oxa‐2,8‐diazaspiro[4.5]dec‐2‐ene‐4,8‐dicarboxylate **7a** (50 mg, 0.13 mmol, 1 equiv.) in dichloromethane (2 mL) was added trifluoroacetic acid (20 µL, 0.26 mmol, 2 equiv.) and the solution stirred for 22 hours. The mixture was quenched with saturated aqueous sodium bicarbonate (20 mL) and extracted with dichloromethane (3 × 7 mL). The combined organic extracts were dried (MgSO_4_), filtered and solvent removed in vacuo. The crude mixture was purified by flash column chromatography (Pet. Ether 40–60/EtOAc, 1:0 to 1:1 followed by DCM/MeOH, 9:1) to yield the pure product, ethyl 3‐phenyl‐1‐oxa‐2,8‐diazaspiro[4.5]dec‐2‐ene‐4‐carboxylate **17a** as a white amorphous solid (26 mg, 0.09 mmol, 68 %); *R*_f_: 0.14 (DCM/MeOH, 9:1); IR (thin film) ν̃ /cm^–1^: 3385 (N‐H, w), 2967 (C‐H, m), 1732 (C=O ester, s), 1671 (C=O carbamate/C=N, s), 1181 (C‐O, s); ^1^H NMR (400 MHz, CDCl_3_, 25 °C) *δ* /ppm: 7.62 (m, 2H), 7.42 (m, 3H), 4.21 (q, *J* = 7.2 Hz, 2H), 4.12 (m, 1H), 3.51–3.28 (m, 4H), 2.34–2.08 (m, 4H), 2.04 (s, 1H), 1.24 (t, *J* = 7.2 Hz, 3H); ^13^C NMR (100 MHz, CDCl_3_) *δ* /ppm: 167.0, 155.7, 130.9, 129.1, 128.5, 126.7, 84.0, 62.5, 62.0, 41.0, 40.7, 33.2, 28.1, 14.1; HRMS (ESI) C_16_H_21_N_2_O_3_ *m/z*: [M + H]^+^ 289.1533 (calc. 289.1547).

**2‐(*tert*‐Butyl) 8‐Ethyl 7‐Benzyl‐2,5,6,7‐tetraazaspiro[3.4]oct‐5‐ene‐2,8‐dicarboxylate (8):** *tert*‐Butyl 3‐(2‐ethoxy‐2‐oxoethylidene) azetidine‐1‐carboxylate **5b** (386 mg, 1.60 mmol, 1.0 equiv.) was dissolved in benzyl azide (2 mL, 16.0, 10.0 equiv.) and heated in a sealed tube at 100 °C for 48 h. This was purified via column chromatography (30 % ethyl acetate in pet. ether) to give the product, 2‐(*tert*‐butyl) 8‐ethyl 7‐benzyl‐2,5,6,7‐tetraazaspiro[3.4]oct‐5‐ene‐2,8‐dicarboxylate **8**, as a yellow oil (204 mg, 0.54 mmol, 34 %); *R*_f_: 0.49 (Pet. Ether 40–60/EtOAc, 7:3); IR (thin film) ν̃ /cm^–1^: 2988 (C‐H, w), 1726 (C=O, s), 1631 (C=C, m), 1115 (C‐O, s); ^1^H NMR (400 MHz, CDCl_3_) *δ*/ppm: 7.30–7.17 (m, 5H), 5.68 (br s, 2H), 4.89 (br s, 2H), 4.41 (q, *J* = 7.0 Hz, 2H), 2.65 (br s, 3H), 1.42–1.38 (m, 12H); ^13^C NMR (101 MHz, CDCl_3_) *δ*/ppm: 154.3, 138.1, 128.9, 128.2, 127.6, 126.8, 89.5, 79.8, 59.0, 54.8, 51.1, 50.3, 48.4, 28.4, 14.6; HRMS (ESI) C_19_H_26_N_4_O_4_Na *m/z*: [M + Na]^+^ 397.1840 (calc. 397.1846).

***tert*‐Butyl 3‐Oxo‐1,2,8‐triazaspiro[4.5]decane‐8‐carboxylate (9):** To *tert*‐butyl 4‐(2‐ethoxy‐2‐oxoethylidene)piperidine‐1‐carboxylate **5a** (270 mg, 1.00 mmol, 1.0 equiv.) in ethanol (0.4 mL) was added hydrazine hydrate (58 µL, 1.2 mmol, 1.2 equiv.) and the mixture heated in a sealed tube at 70 °C for 24 hours. Solvent was removed in vacuo and the mixture purified by flash column chromatography (DCM/MeOH, 19:1 to 9:1) to yield the product, *tert*‐butyl 3‐oxo 1,2,8‐triazaspiro[4.5]decane‐8‐carboxylate **9**, as a colourless oil (173 mg, 0.68 mmol, 68 %); *R*_f_: 0.30 (DCM/MeOH, 9:1); IR (thin film) ν̃ /cm^–1^: 3225 (N‐H, m), 2931 (C‐H, m), 1668 (C=O carbamate and acid hydrazide, br s), 1148 (C‐O, s); ^1^H NMR (400 MHz, CDCl_3_, 25 °C) *δ* /ppm: 7.12 (br s, 1H), 4.03 (br s, 1H), 3.55–3.37 (m, 4H), 2.34 (s, 2H), 1.79–1.60 (m, 4H), 1.45 (s, 9H); ^13^C NMR (100 MHz, CDCl_3_) *δ* /ppm: 176.8, 154.6, 79.8, 61.1, 43.0, 40.2, 34.5, 28.4; HRMS (ESI) C_12_H_21_N_3_O_3_Na *m/z*: [M + Na]^+^ 278.1479 (calc. 278.1475).

***tert*‐Butyl 4‐[(4‐Methoxyphenyl)imino]piperidine‐1‐carboxylate (6):** To a solution of *tert*‐butyl 4‐oxopiperidine‐1‐carboxylate **4a** (0.50 g, 2.5 mmol, 1.0 equiv.) in toluene (10 mL) was added *para*‐anisidine (0.31 g, 2.5 mmol, 1.0 equiv.) and the mixture heated to vigorous reflux in Dean–Stark apparatus. After 20 hours, the mixture was cooled and solvent removed in vacuo to yield the crude product, which was used directly in the following reaction, without purification; *R*_f_: 0.33 (Pet. Ether 40–60/EtOAc, 2:3); IR (thin film) ν̃ /cm^–1^: 3357 (Ar‐H, w), 2976 (C‐H, m), 1685 (C=O carbamate and imine, s), 1510 (C=C Ar, s), 1234 (C‐O, s), 1160 (C‐O, s); ^1^H NMR (400 MHz, CDCl_3_, 25 °C) *δ* /ppm: 6.84 (d, *J* = 8.8 Hz, 2H), 6.64 (d, *J* = 8.8 Hz, 2H), 3.78 (s, 3H), 3.69 (m, 2H), 3.48 (m, 2H), 2.54 (m, 1H), 2.43 (m, 1H), 2.33 (m, 2H), 1.47 (br s, ′9H′); HRMS (ESI) C_17_H_25_N_2_O_3_ *m/z*: [M + H]^+^ 305.1874 (calc. 305.1865).

***tert*‐Butyl 3‐Acetoxy‐1‐(4‐methoxyphenyl)‐2‐oxo‐1,7‐diazaspiro[3.5]nonane‐7‐carboxylate (10):** To a solution of crude product, containing *tert*‐butyl 4‐[(4‐methoxyphenyl)imino]piperidine‐1‐carboxylate **6** (250 mg, 0.82 mmol, 1 equiv.), in dichloromethane (6 mL) at –78 °C was added triethylamine (0.34 mL, 2.46 mmol, 3 equiv.) dropwise. A solution of acetoxyacetyl chloride (0.22 mL, 1.98 mmol, 2.4 equiv.) in dichloromethane (2 mL) was added dropwise over 30 minutes and the solution was warmed slowly to room temperature and stirred for 20 hours. The mixture was washed with sodium bicarbonate solution (5 %, 10 mL), HCl (5 %, 10 mL) and saturated aqueous sodium chloride (10 mL), dried (MgSO_4_), filtered and the solvent was removed in vacuo. The mixture was purified by flash column chromatography (Pet. Ether 40–60/EtOAc, 9:1 to 4:6) to yield the product, *tert*‐butyl 3‐acetoxy‐1‐(4‐methoxyphenyl)‐2‐oxo‐1,7‐diazaspiro[3.5]nonane‐7‐carboxylate **10**, as a pale yellow oil (183 mg, 0.45 mmol, 55 %); *R*_f_: 0.48 (Pet. Ether 40–60/EtOAc, 4:6); IR (thin film) ν̃ /cm^–1^: 3319 (Ar‐H, w), 2975 (C‐H, m), 1750 (C=O ester and lactam, s), 1687 (C=O carbamate, s), 1511 (C=C Ar, s), 1215 (C‐O, s), 1163 (C‐O, s); ^1^H NMR (400 MHz, CDCl_3_, 25 °C) *δ* /ppm: 7.35 (d, *J* = 9.1 Hz, 2H), 6.87 (d, *J* = 9.1 Hz, 2H), 5.72 (s, 1H), 4.13 (m, 2H), 3.79 (s, 3H), 3.50 (m, 1H), 3.01 (m, 1H), 2.58 (m, 1H), 2.27 (m, 1H), 2.20 (s, 3H), 2.05 (m, 1H), 1.81 (m, 1H), 1.65 (m, 1H), 1.46 (s, ′9H′); ^13^C NMR (100 MHz, CDCl_3_) *δ* /ppm: 169.8, 162.2, 157.6, 154.7, 128.5, 122.3, 114.7, 80.8, 80.3, 79.5, 66.0, 52.1, 55.6, 33.5, 31.1, 28.5, 20.9; HRMS (ESI) C_21_H_29_N_2_O_6_ *m/z*: [M + H]^+^ 405.2019 (calc. 405.2026).

## Supporting information

Supporting Information
