# Supplementary material for: Cycloaddition Strategies for the Synthesis of Diverse Heterocyclic Spirocycles for Fragment‐Based Drug Discovery
Source: European J Org Chem. 2019 Jul 29;2019(31-32):5219–29. doi: 10.1002/ejoc.201900847 (PMC6774287; doi:10.1002/ejoc.201900847)
Supplement: Supplementary file 1 — Supporting Information [file EJOC-2019-5219-s001.pdf]

European Journal of Organic Chemistry

Supporting Information

Cycloaddition Strategies for the Synthesis of Diverse Heterocyclic Spirocycles for Fragment-Based Drug Discovery

Thomas A. King, Hannah L. Stewart, Kim T. Mortensen,
Andrew J. P. North, Hannah F. Sore, and David R. Spring*

ejoc201900847-sup-0001-SupMat.pdf

Contents

1. Compounds Properties 2
2. NMR Spectra 3
N-hydroxybenzimidoyl chloride (12) 3
Ethyl (tert-butoxycarbonyl)-l-serinate (15) 4
Ethyl 2-((tert-butoxycarbonyl)amino)acrylate (1) 5
tert-Butyl 4-(2-ethoxy-2-oxoethylidene) piperidine-1-carboxylate (5a) 6
..... 6
tert-butyl 3-(2-ethoxy-2-oxoethylidene) azetidine-1-carboxylate (5b) 7
tert-Butyl 3-(2-ethoxy-2-oxoethylidene)pyrrolidine-1-carboxylate (5c) 8
tert-Butyl 3-(2-ethoxy-2-oxoethylidene)azepane-1-carboxylate (5e) 12
Ethyl 4-((tert-butoxycarbonyl)amino)-3-phenyl-4,5-dihydroisoxazole-4-carboxylate (2) 13
tert-Butyl (4-(hydroxymethyl)-3-phenyl-4,5-dihydroisoxazol-4-yl)carbamate (2') 14
3-Phenyl-1,8-dioxa-2,6-diazaspiro[4.4]non-2-en-7-one (3) 15
8-(tert-Butyl) 4-ethyl 3-phenyl-1-oxa-2,8-diazaspiro[4.5]dec-2-ene-4,8-dicarboxylate (7a) 16
2-(tert-Butyl) 8-ethyl 7-phenyl-5-oxa-2,6-diazaspiro[3.4]oct-6-ene-2,8-dicarboxylate (7b) 17
7-(tert-Butyl) 4-ethyl 3-phenyl-1-oxa-2,7-diazaspiro[4.4]non-2-ene-4,7-dicarboxylate (7c) 18
7-(tert-Butyl) 4-ethyl 3-phenyl-1-oxa-2,7-diazaspiro[4.5]dec-2-ene-4,7-dicarboxylate (7d) 19
7-(tert-Butyl) 4-ethyl 3-phenyl-1-oxa-2,7-diazaspiro[4.6]undec-2-ene-4,7-dicarboxylate (7e) 20
Ethyl 3-phenyl-1-oxa-2,8-diazaspiro[4.5]dec-2-ene-4-carboxylate (17a) 21
2-(tert-Butyl) 8-ethyl 7-benzyl-2,5,6,7-tetraazaspiro[3.4]oct-5-ene-2,8-dicarboxylate (8) 22
tert-Butyl 3-oxo-1,2,8-triazaspiro[4.5]decane-8-carboxylate (9) 23
tert-Butyl 4-((4-methoxyphenyl)imino)piperidine-1-carboxylate (6) 24
tert-Butyl 3-acetoxy-1-(4-methoxyphenyl)-2-oxo-1,7-diazaspiro[3.5]nonane-7-carboxylate (10) 25
3. X-ray Crystallography 26
3-Phenyl-1,8-dioxa-2,6-diazaspiro[4.4]non-2-en-7-one (3) 26

1. Compounds Properties

Calculated using Data Warrior

Compound	MW	cLogP	HAC	HBA	HBD	pTSA	Shape Index	Molecular Complexity	Chiral Centres	RBC	sp^3-Atom
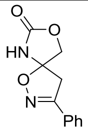	218	1.98	16	5	1	59.9	0.625	0.755	1	1	5
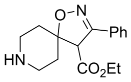	288	2.48	21	5	1	59.9	0.476	0.824	1	4	11
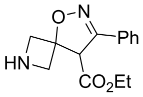	260	1.80	19	5	1	59.9	0.526	0.809	1	4	9
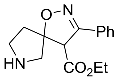	274	2.40	20	5	1	59.9	0.500	0.838	2	4	10
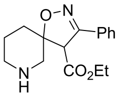	288	2.48	21	5	1	59.9	0.476	0.864	2	4	11
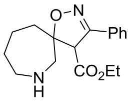	302	2.83	22	5	1	59.9	0.455	0.868	2	4	12
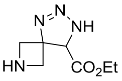	184	-1.43	13	6	2	75.1	0.615	0.680	1	3	8
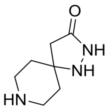	155	-1.76	11	4	3	53.2	0.636	0.645	0	0	8
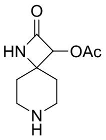	198	-0.83	14	5	2	67.4	0.571	0.699	1	2	9
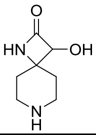	156	-1.31	11	4	3	61.4	0.636	0.685	1	0	8
Average	232	0.866	16.8	4.9	1.6	61.6	0.552	0.767	1.2	2.6	9.1

MW = molecular weight,

PSA = polar surface area,

HBA = number of hydrogen-bond acceptors,

HBD = number of hydrogen-bond donors,

HAC = heavy atom count,

RBC = rotatable bond count.

2. NMR Spectra

N-hydroxybenzimidoyl chloride (12)

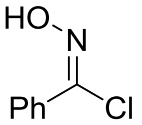

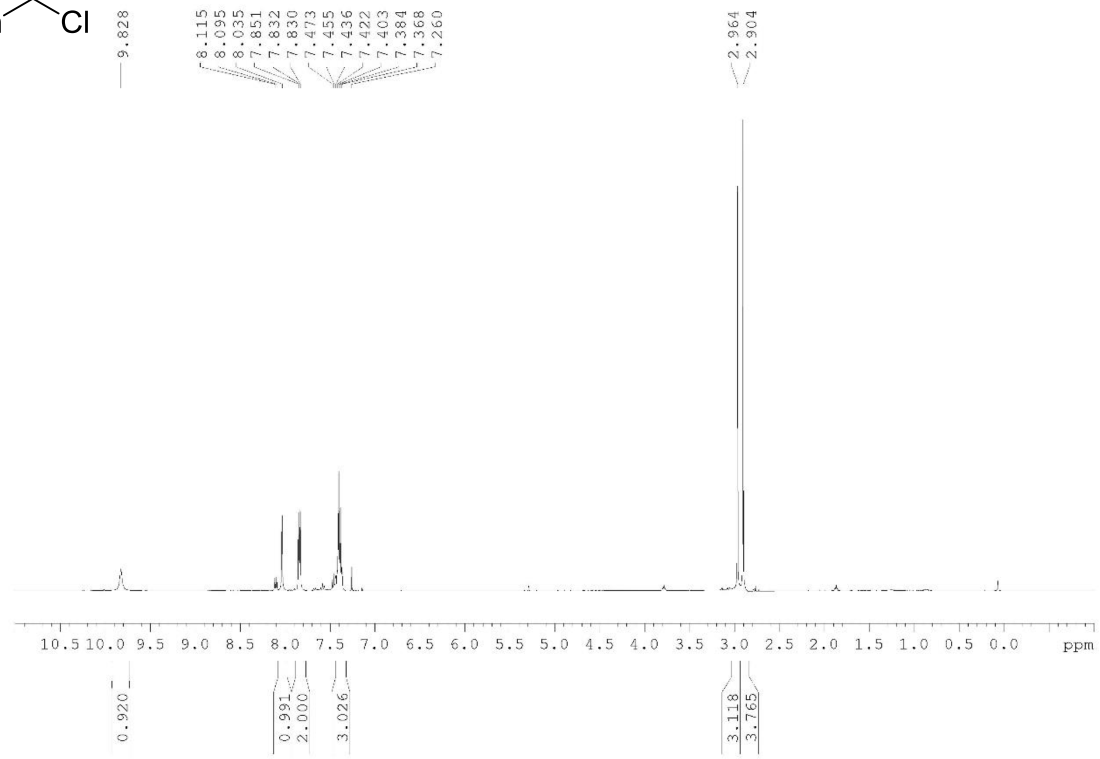

Ethyl (tert-butoxycarbonyl)-l-serinate (15)

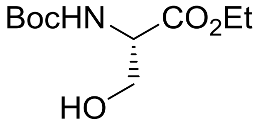

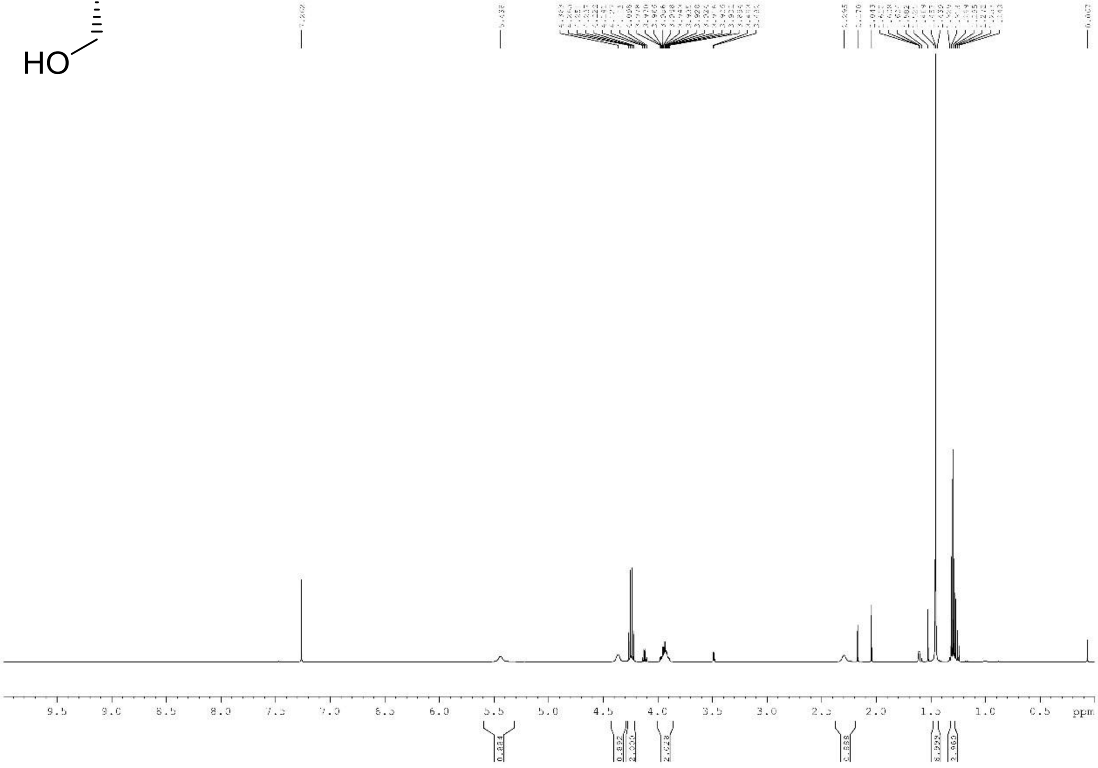

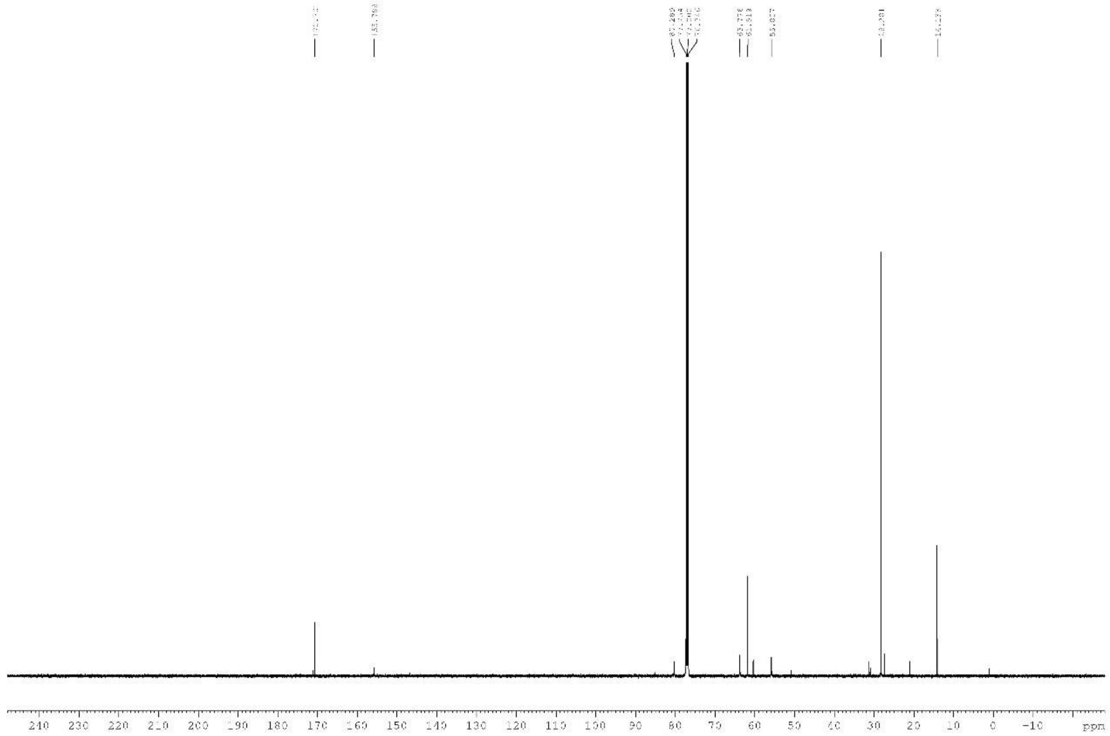

Ethyl 2-((tert-butoxycarbonyl)amino)acrylate (1)

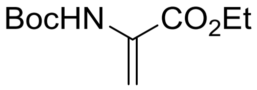

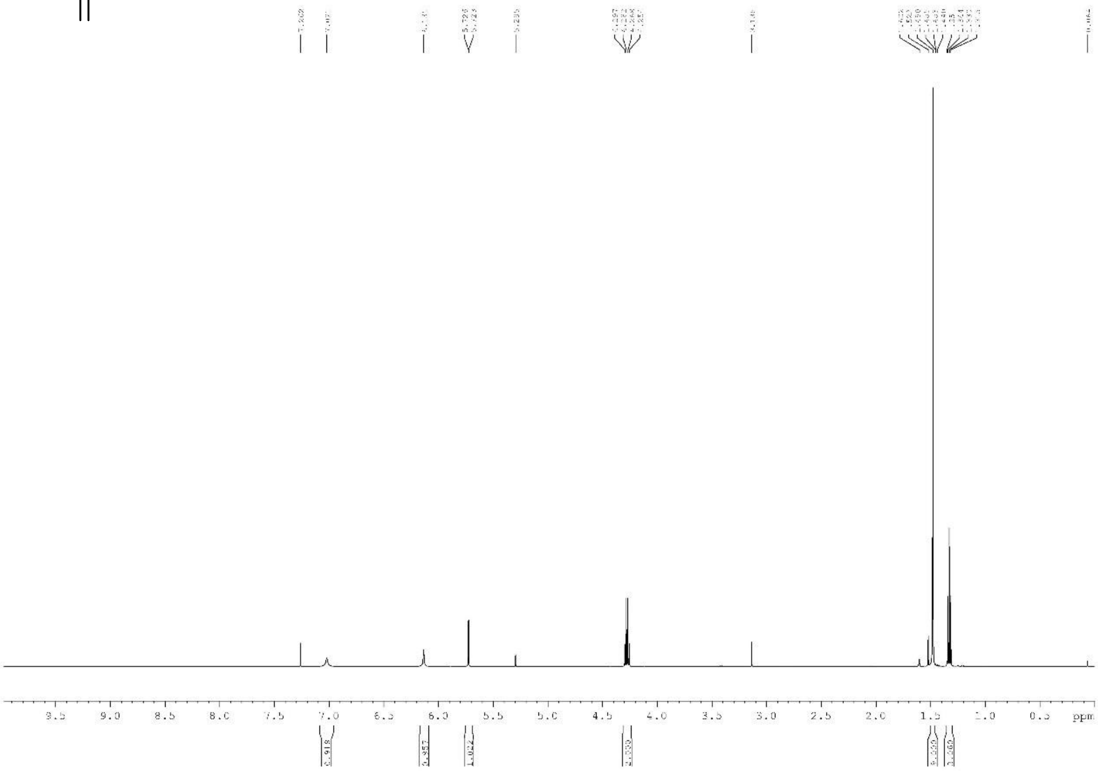

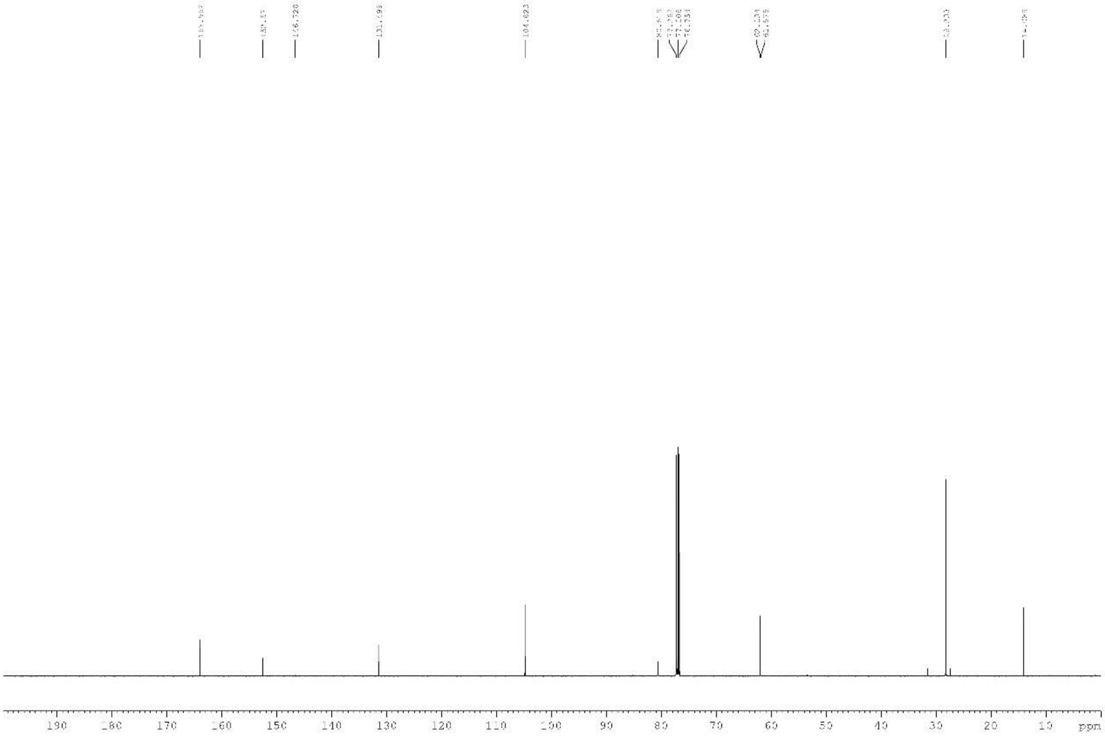

***tert*-Butyl 4-(2-ethoxy-2-oxoethylidene) piperidine-1-carboxylate (5a)**

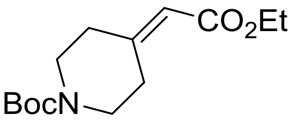

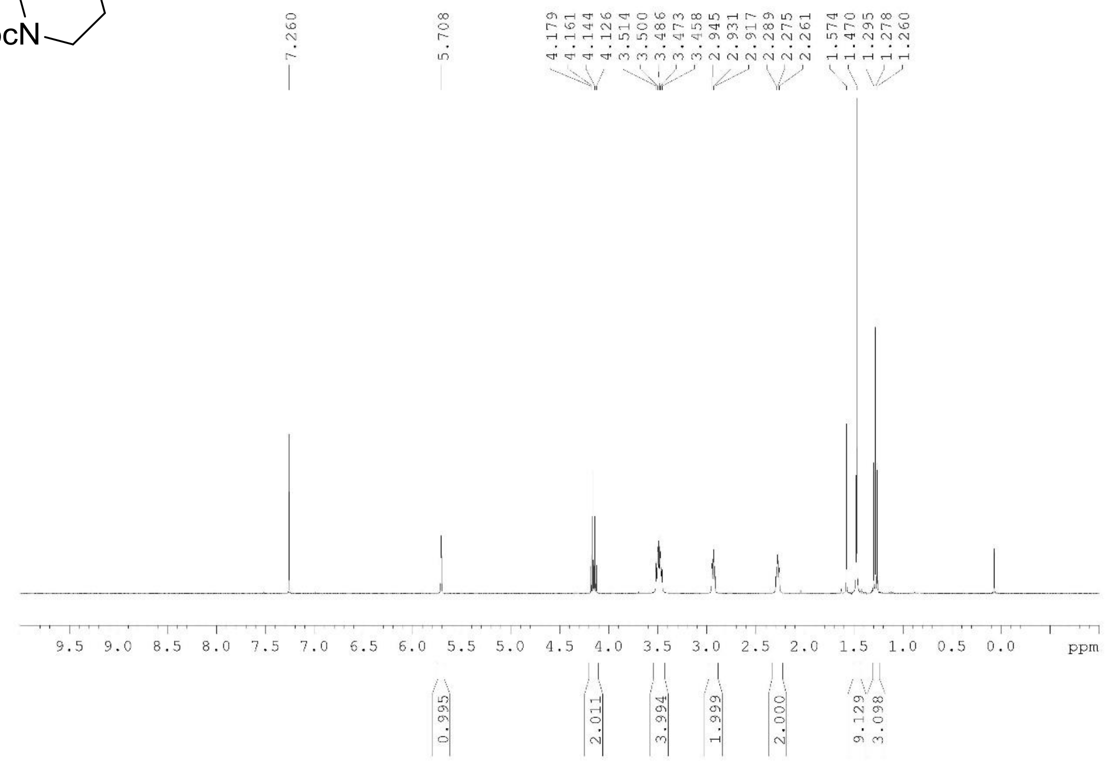

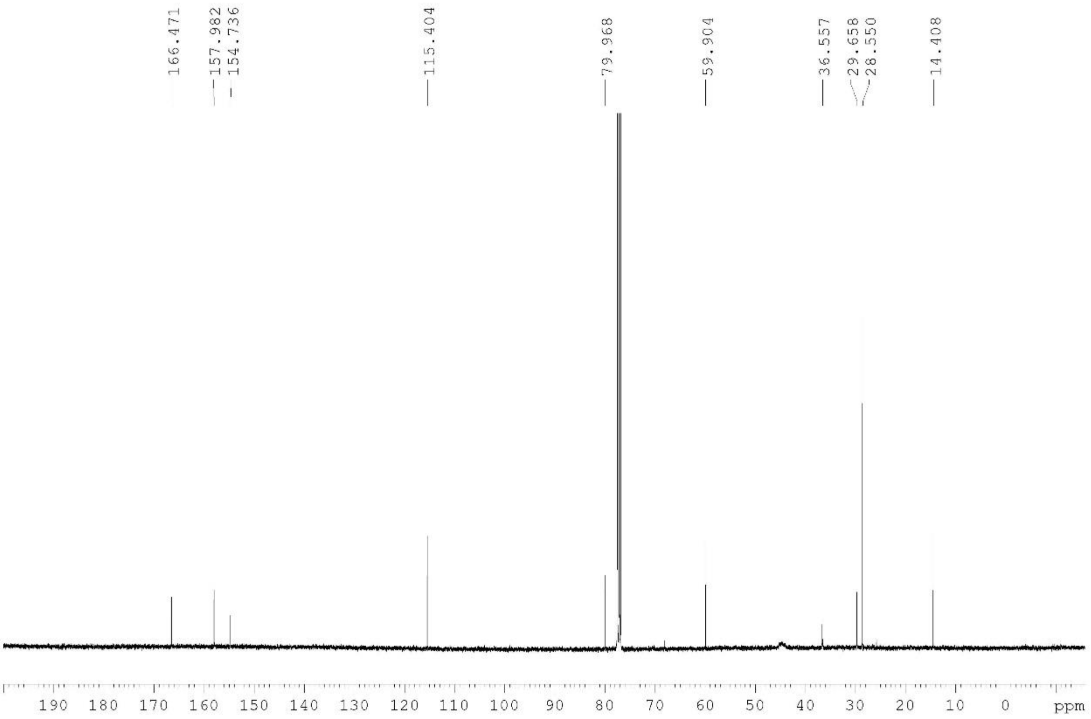

***tert*-butyl 3-(2-ethoxy-2-oxoethylidene) azetidine-1-carboxylate (5b)**

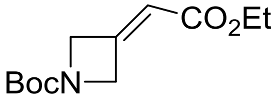

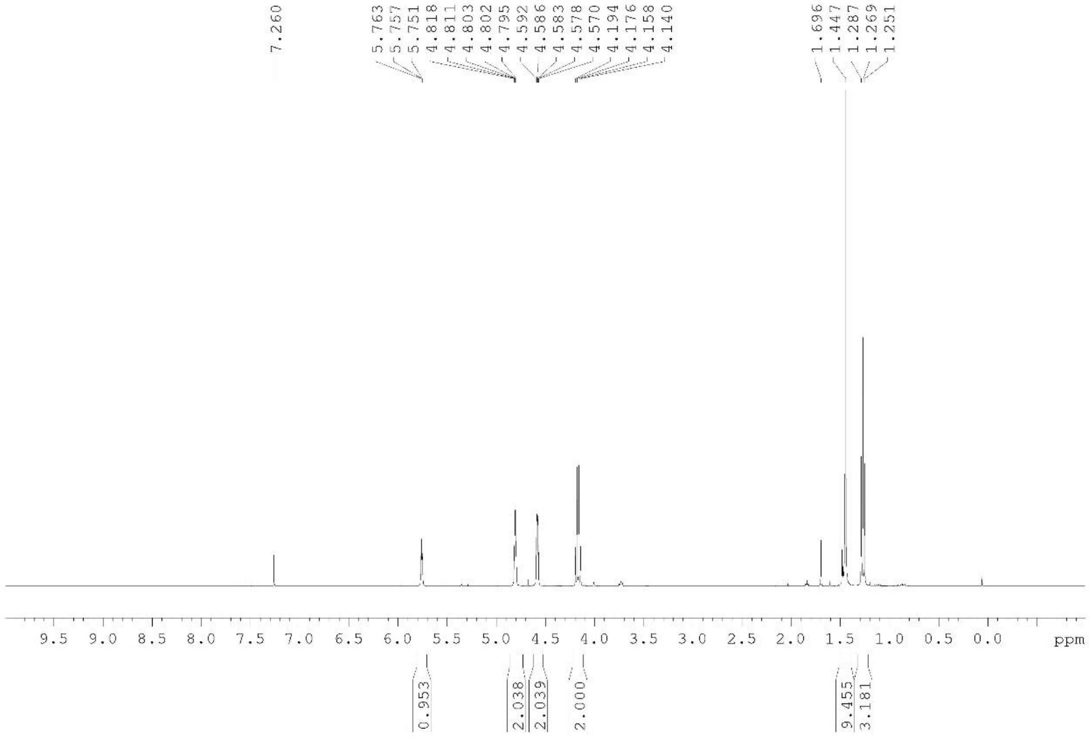

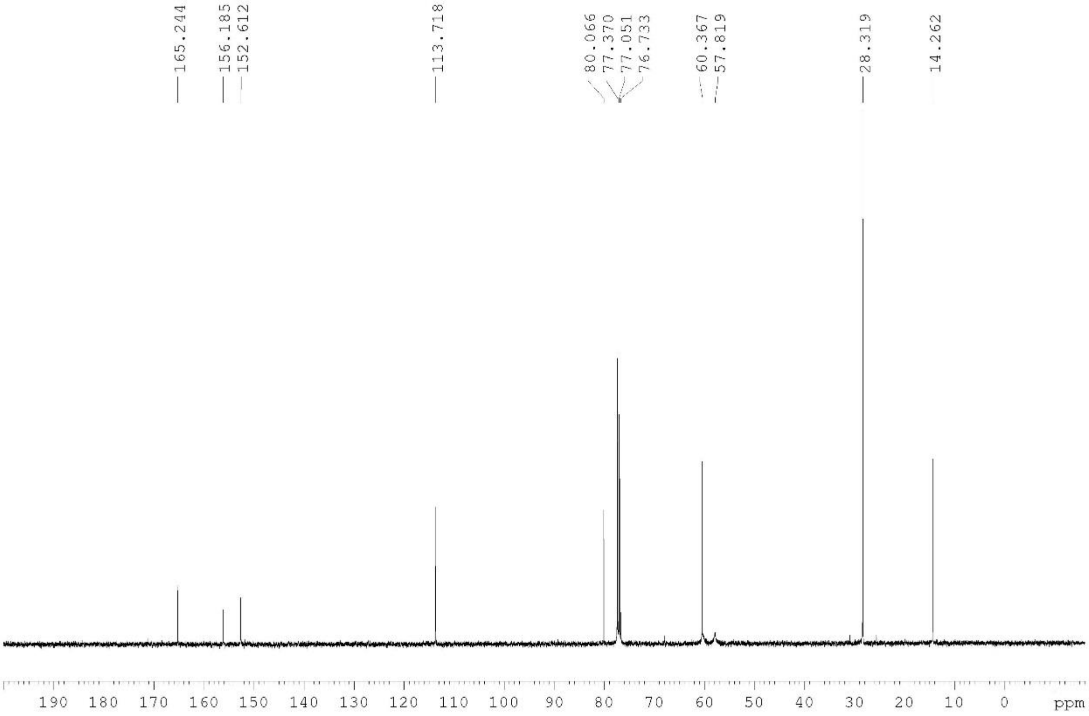

***tert*-Butyl 3-(2-ethoxy-2-oxoethylidene)pyrrolidine-1-carboxylate (5c)**

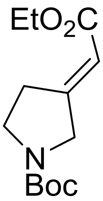

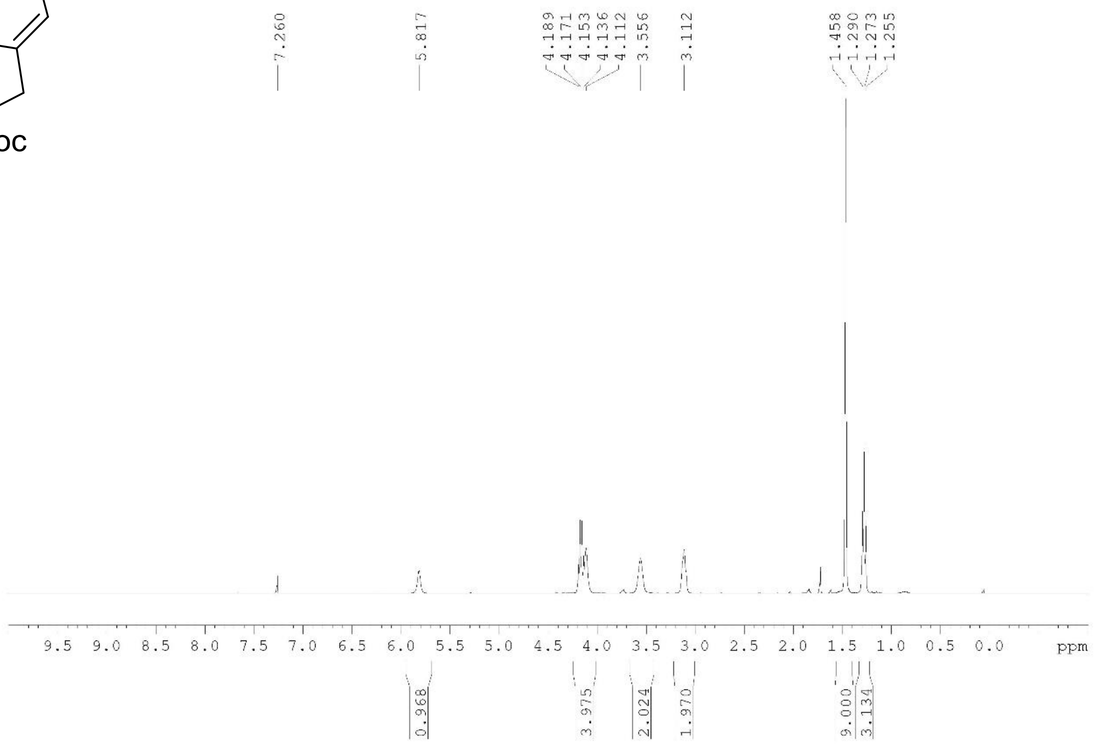

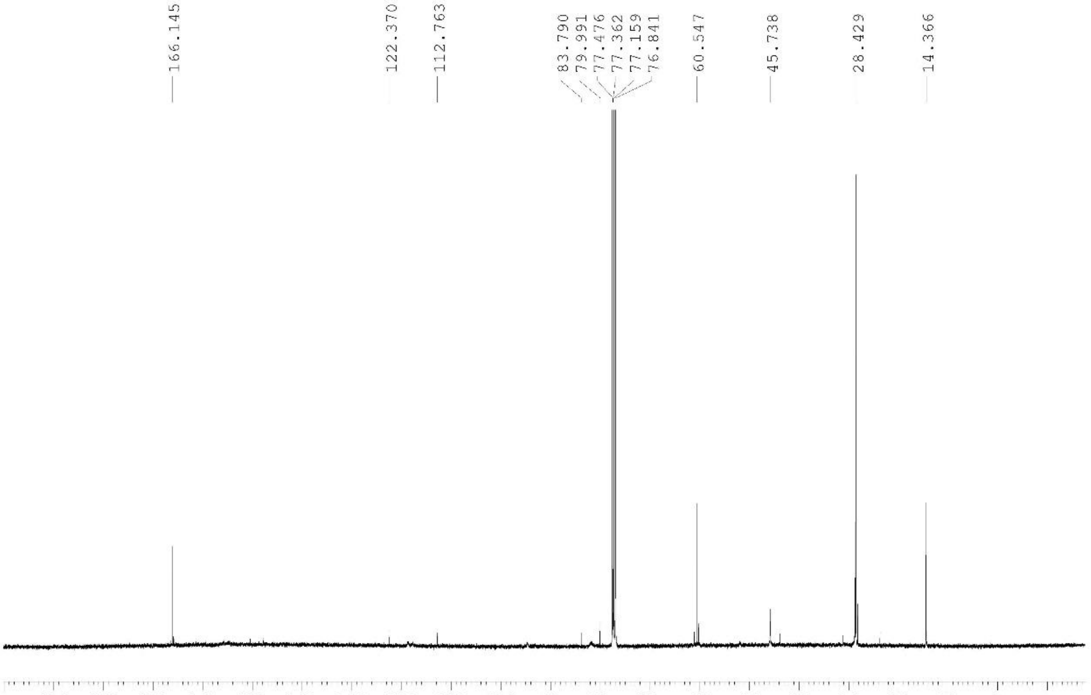

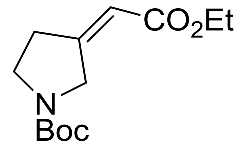

CO2Et
N
Boc

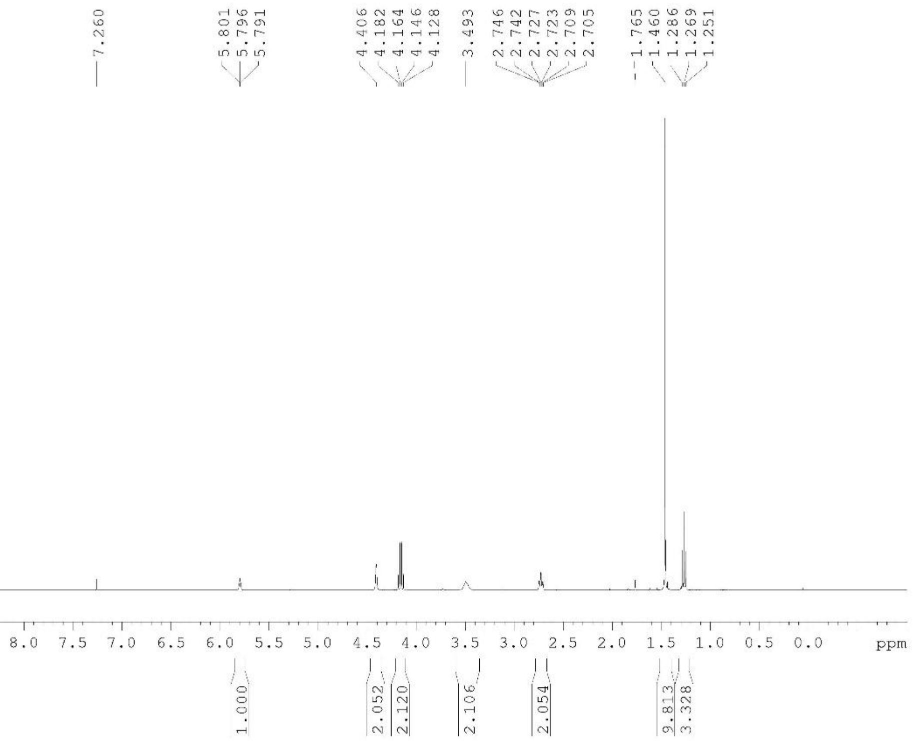

7.260
5.801
5.796
5.791
4.406
4.182
4.164
4.146
4.128
3.493
2.746
2.742
2.727
2.723
2.709
2.705
1.765
1.460
1.286
1.269
1.251
9.5 9.0 8.5 8.0 7.5 7.0 6.5 6.0 5.5 5.0 4.5 4.0 3.5 3.0 2.5 2.0 1.5 1.0 0.5 0.0 ppm
1.000
2.052
2.120
2.106
2.054
9.813
3.328

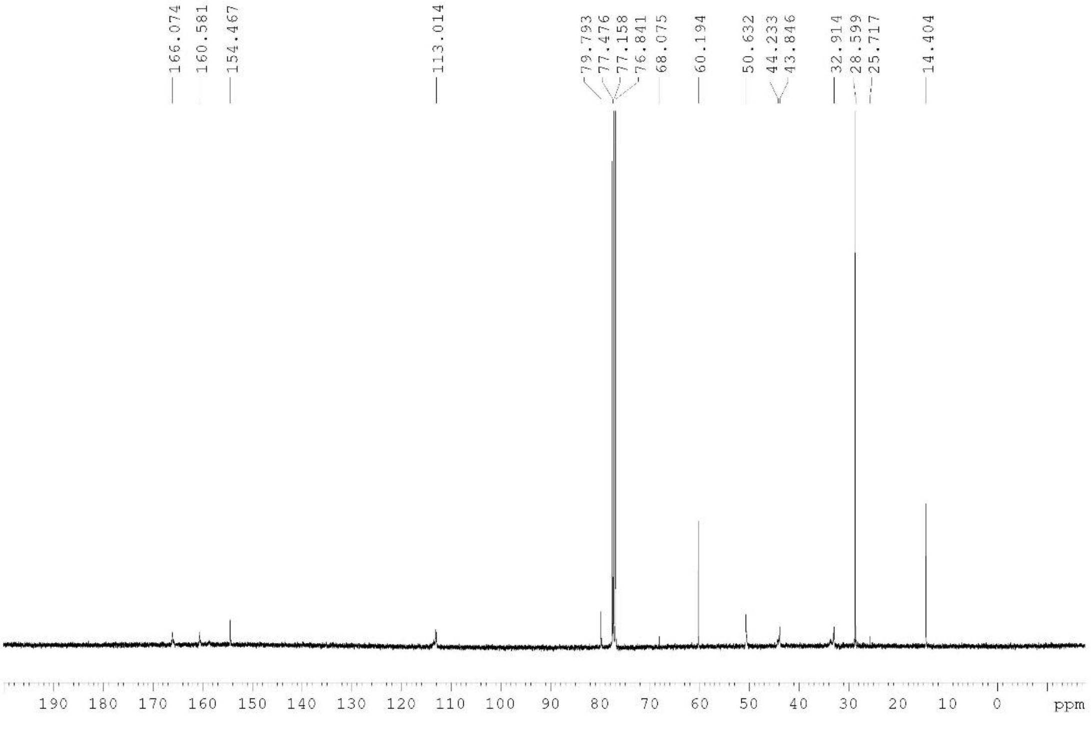

166.074
160.581
154.467
113.014
79.793
77.476
77.158
76.841
68.075
60.194
50.632
44.233
43.846
32.914
28.599
25.717
14.404
190 180 170 160 150 140 130 120 110 100 90 80 70 60 50 40 30 20 10 0 ppm

***tert*-Butyl 3-(2-ethoxy-2-oxoethylidene) piperidine-1-carboxylate (5d)**

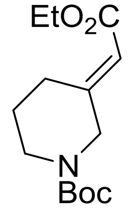

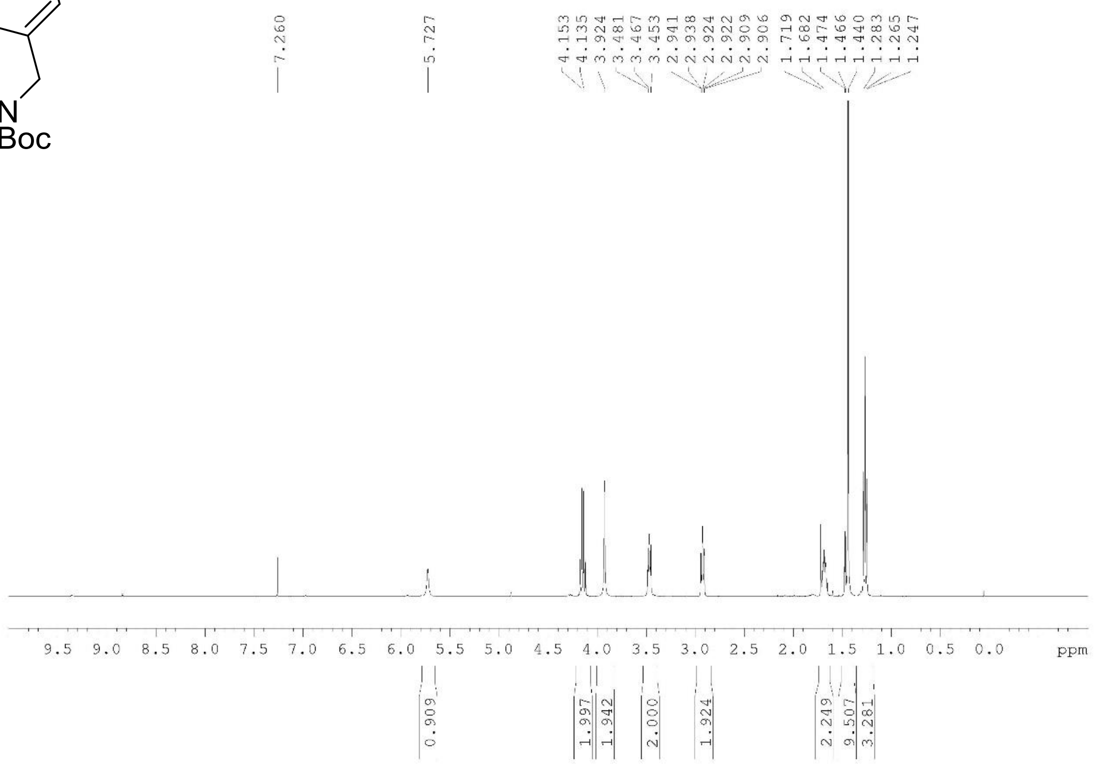

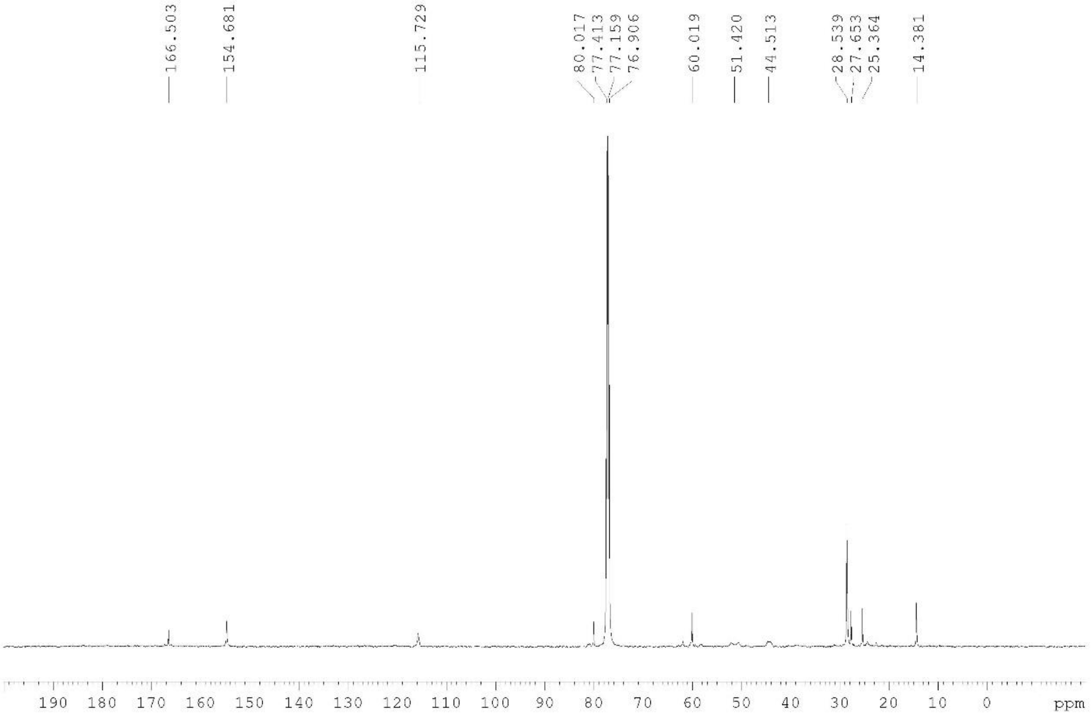

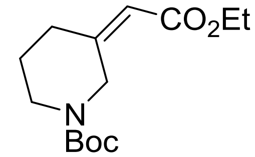
CO2Et
N
Boc

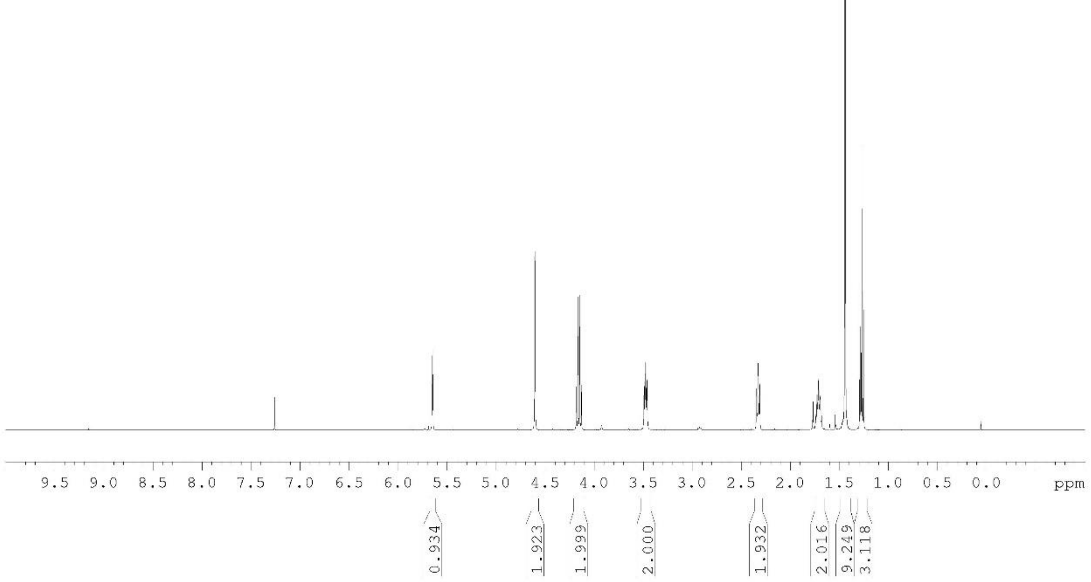
5.652
5.649
5.645
4.604
4.602
4.183
4.165
4.147
4.129
3.489
3.474
3.460
2.344
2.341
2.328
2.326
2.312
2.310
1.710
1.439
1.289
1.271
1.253
9.5 9.0 8.5 8.0 7.5 7.0 6.5 6.0 5.5 5.0 4.5 4.0 3.5 3.0 2.5 2.0 1.5 1.0 0.5 0.0 ppm
0.934
1.923
1.999
2.000
1.932
2.016
9.249
3.118

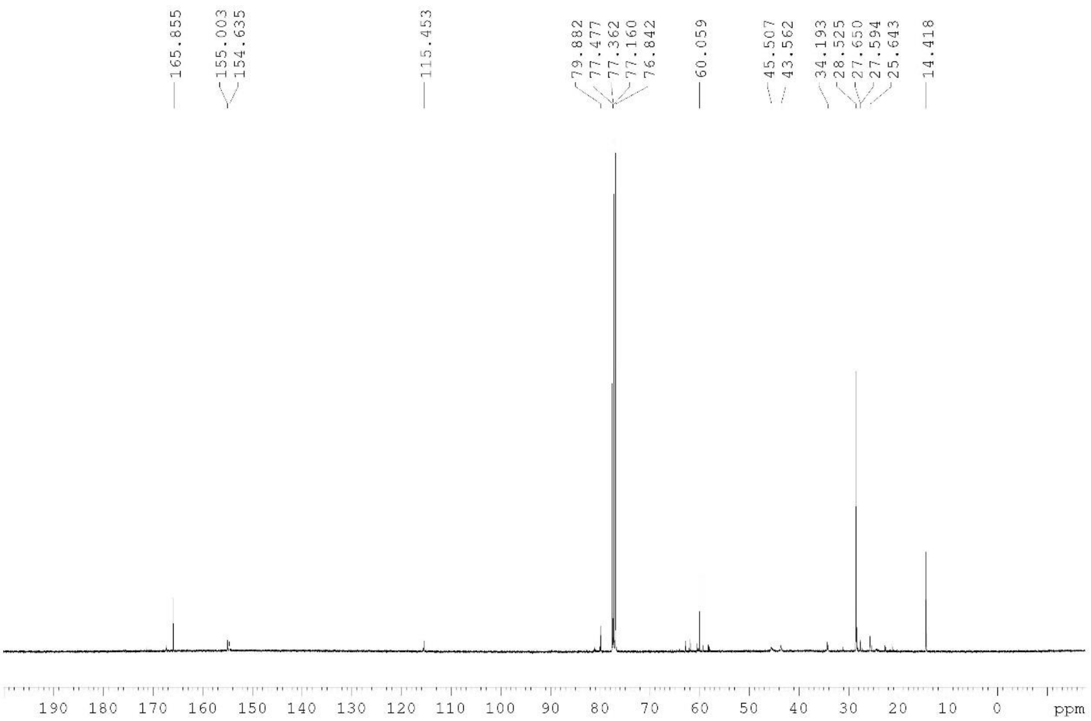
165.855
155.003
154.635
115.453
79.882
77.477
77.362
77.160
76.842
60.059
45.507
43.562
34.193
28.525
27.650
27.594
25.643
14.418
190 180 170 160 150 140 130 120 110 100 90 80 70 60 50 40 30 20 10 0 ppm

***tert*-Butyl 3-(2-ethoxy-2-oxoethylidene)azepane-1-carboxylate (5e)**

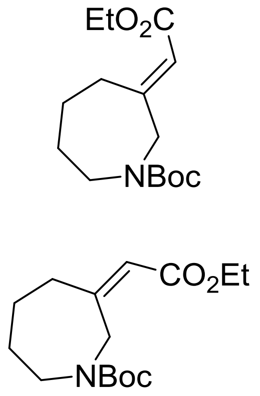

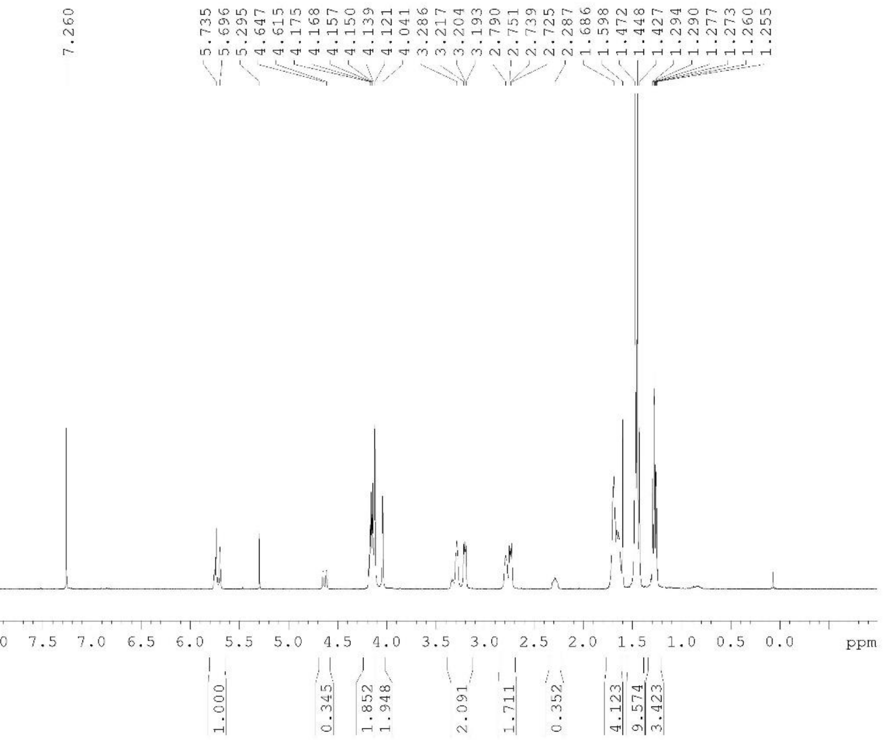

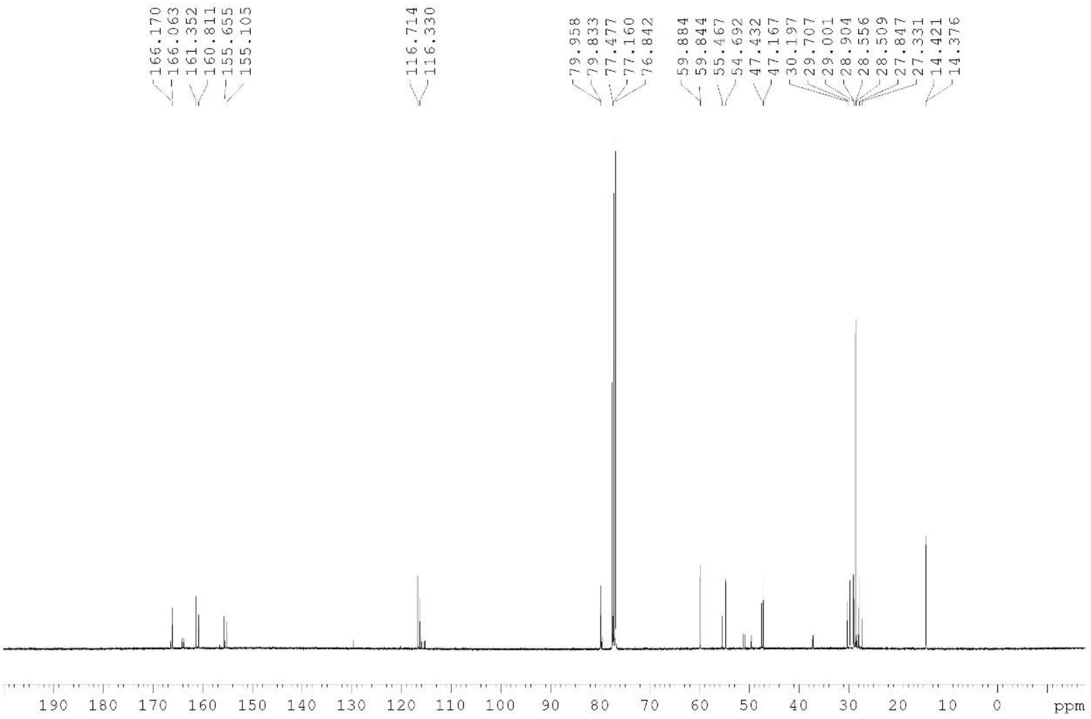

Ethyl 4-((tert-butoxycarbonyl)amino)-3-phenyl-4,5-dihydroisoxazole-4-carboxylate (2)

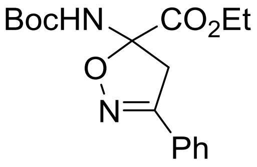

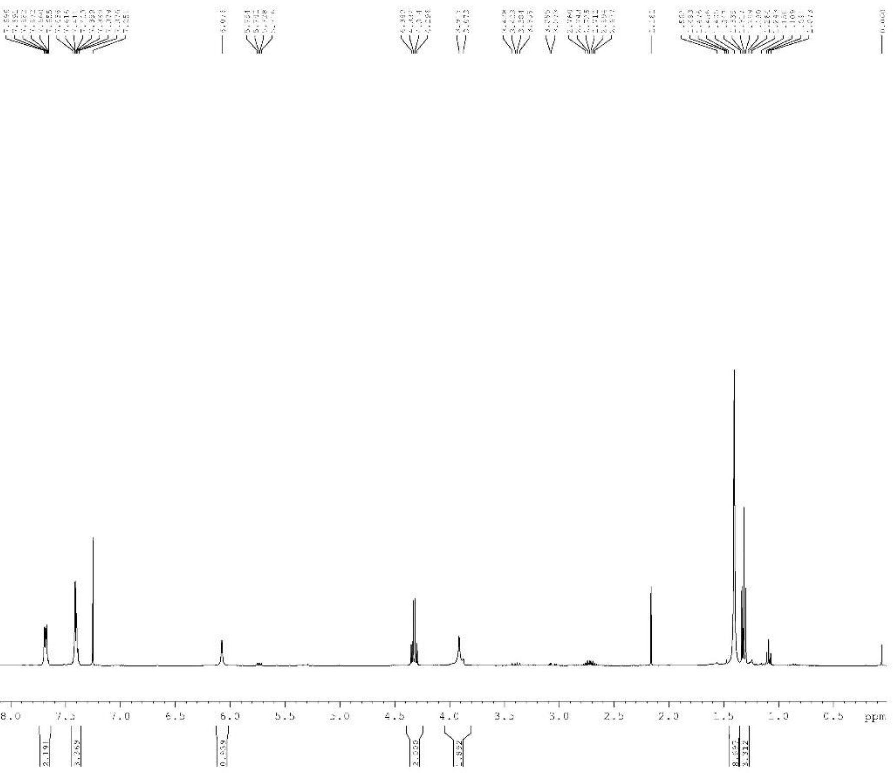

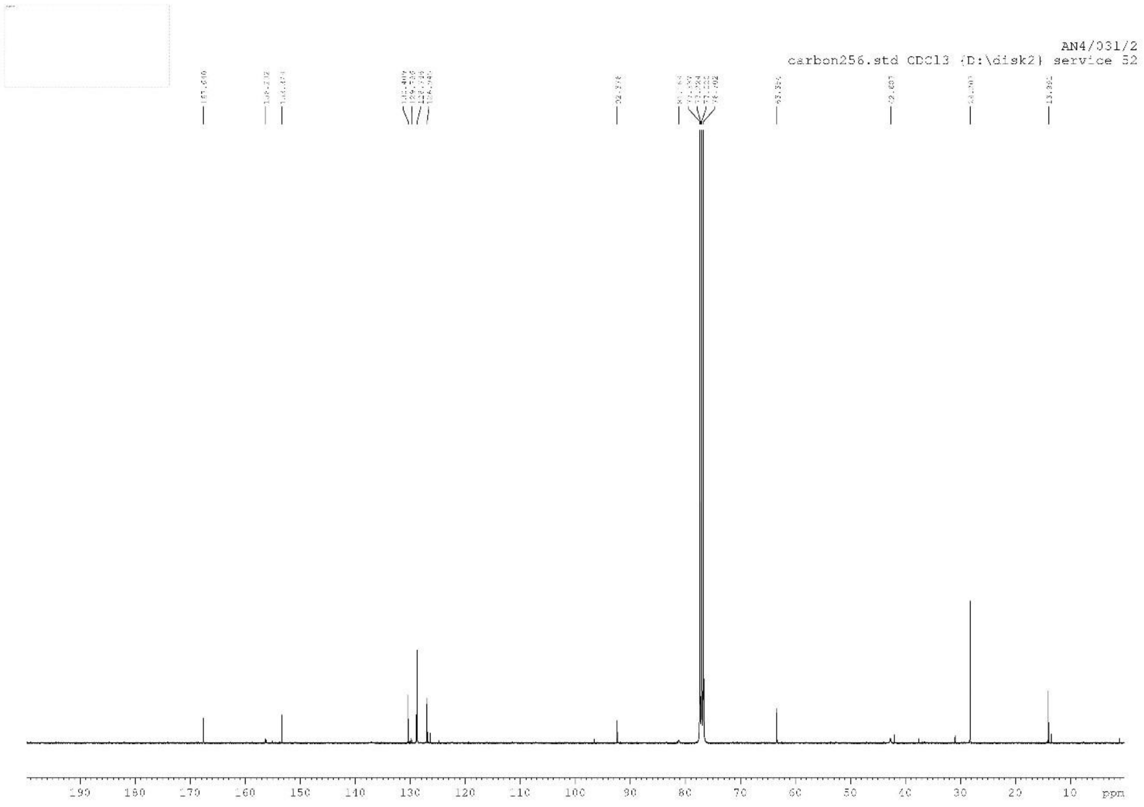

***tert*-Butyl (4-(hydroxymethyl)-3-phenyl-4,5-dihydroisoxazol-4-yl)carbamate (2')**

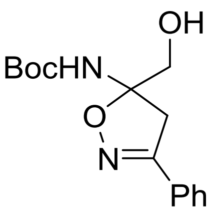

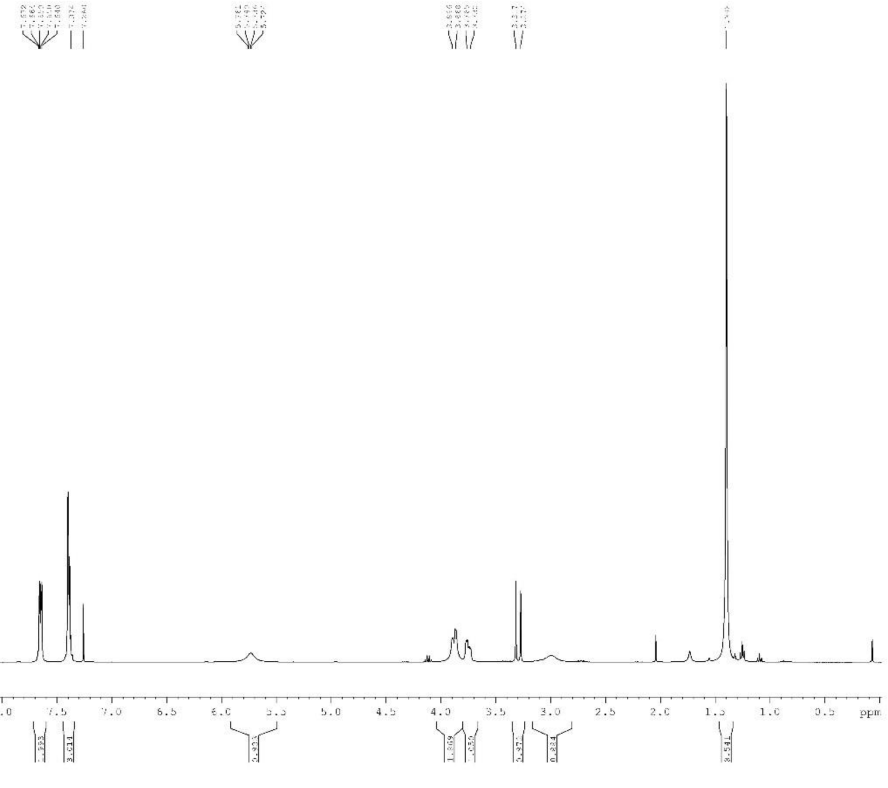

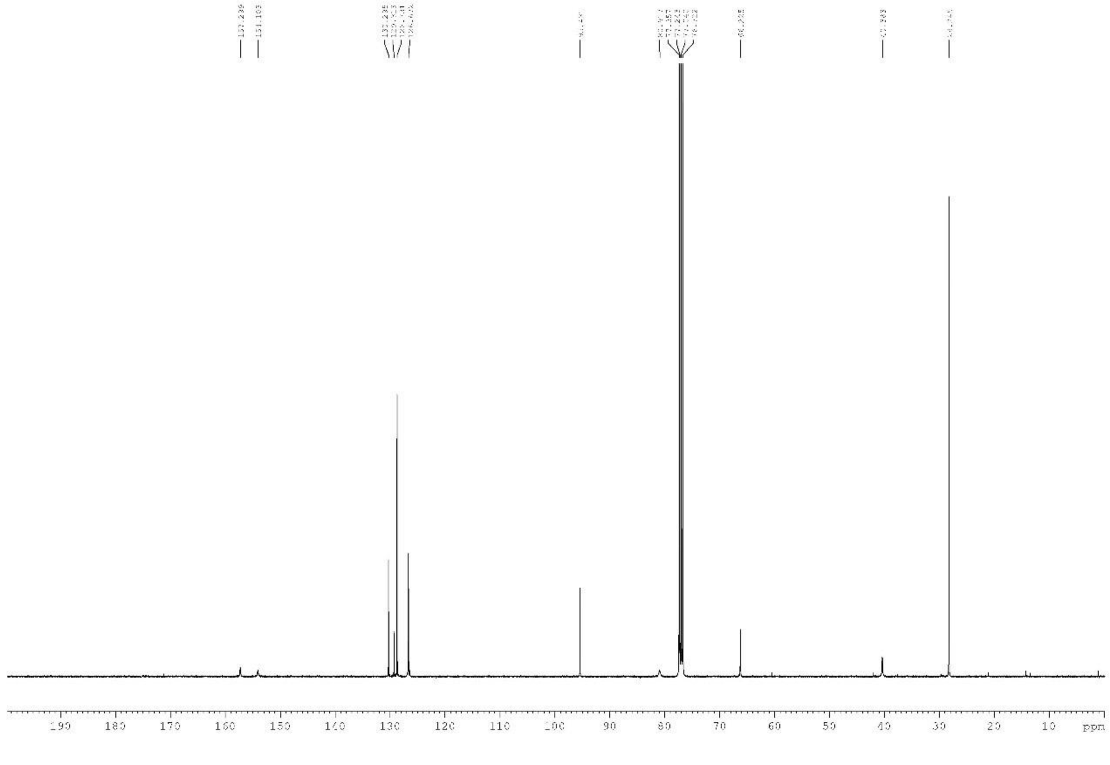

3-Phenyl-1,8-dioxa-2,6-diazaspiro[4.4]non-2-en-7-one (3)

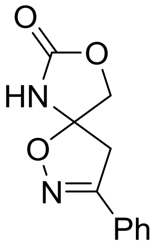

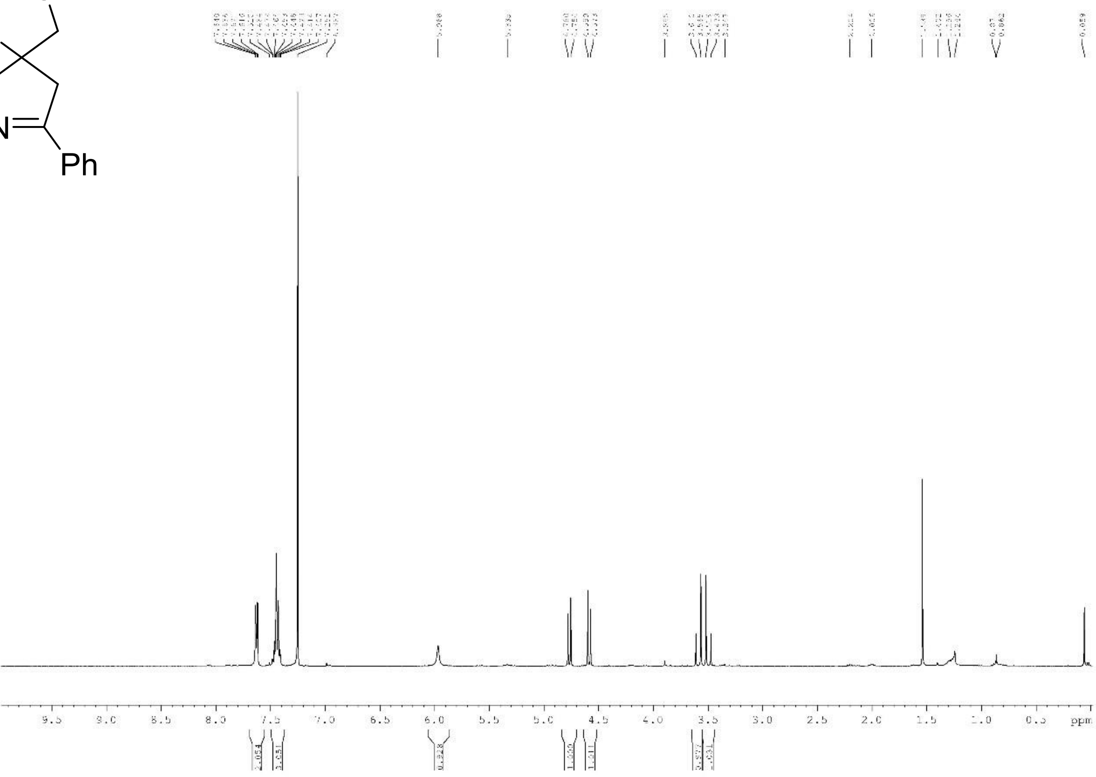

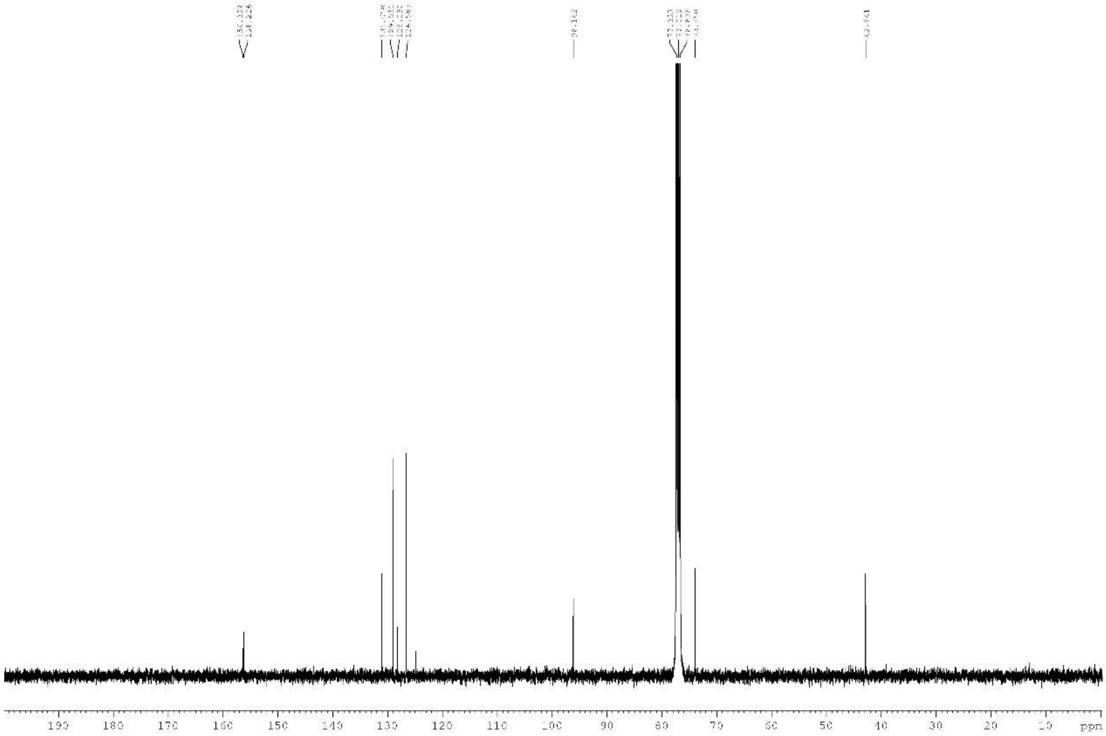

8-(tert-Butyl) 4-ethyl 3-phenyl-1-oxa-2,8-diazaspiro[4.5]dec-2-ene-4,8-dicarboxylate (7a)

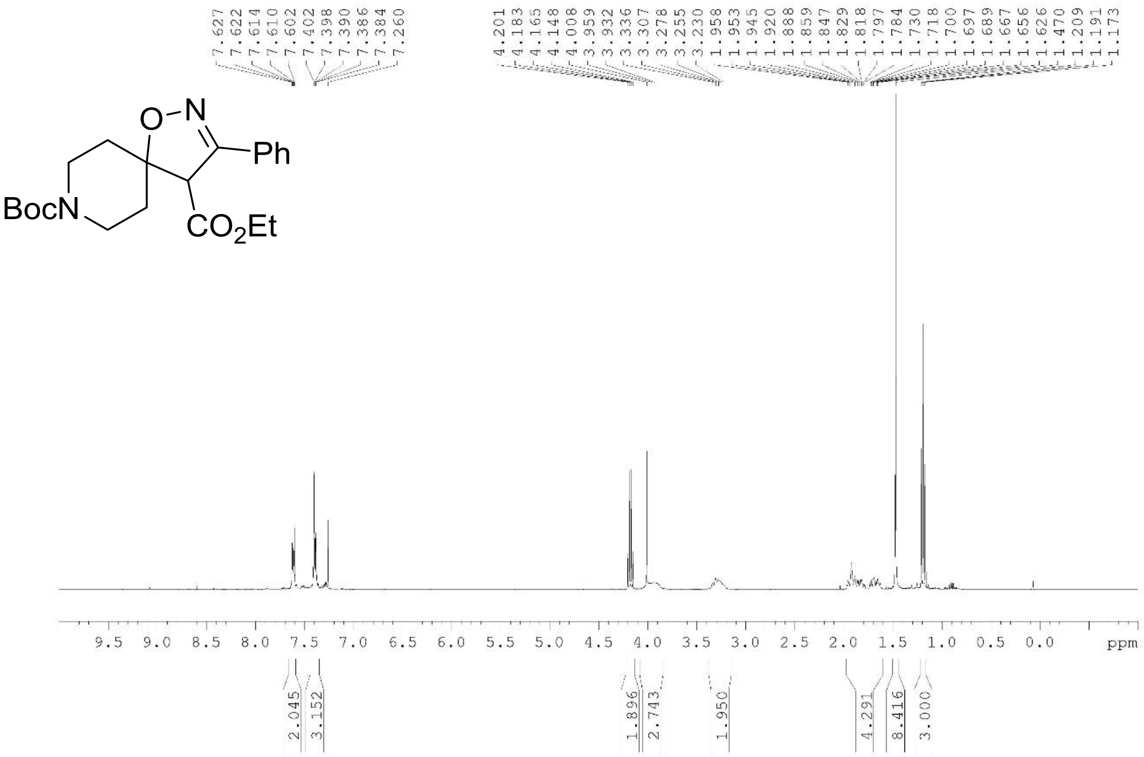

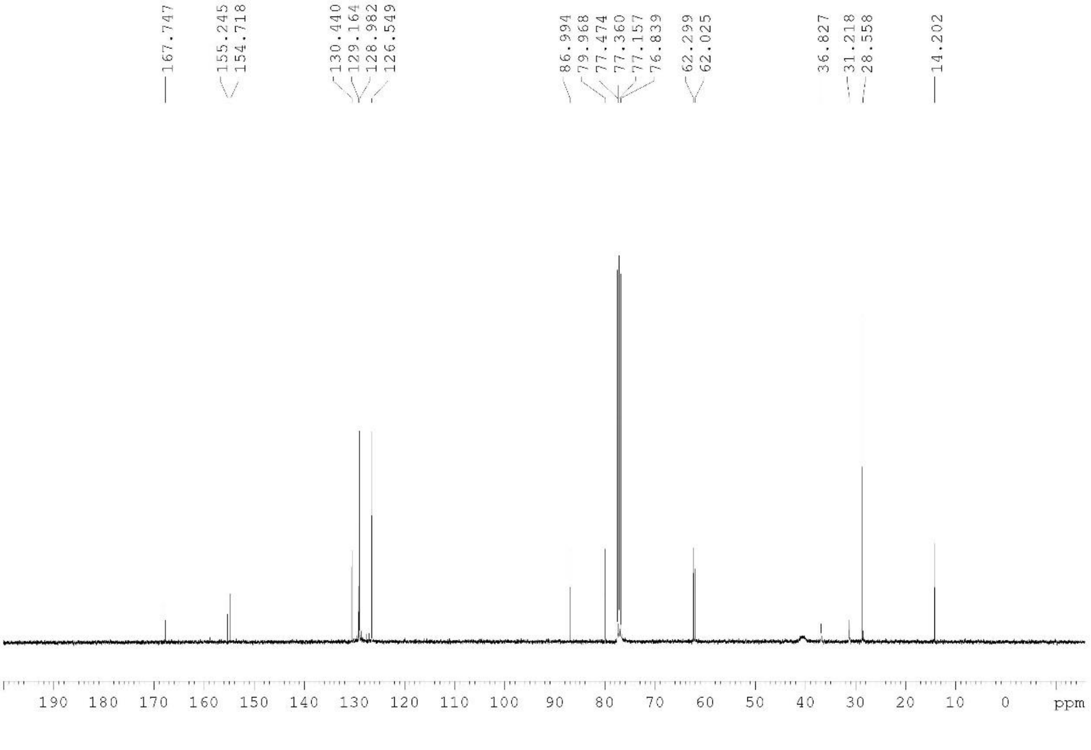

2-(tert-Butyl) 8-ethyl 7-phenyl-5-oxa-2,6-diazaspiro[3.4]oct-6-ene-2,8-dicarboxylate (7b)

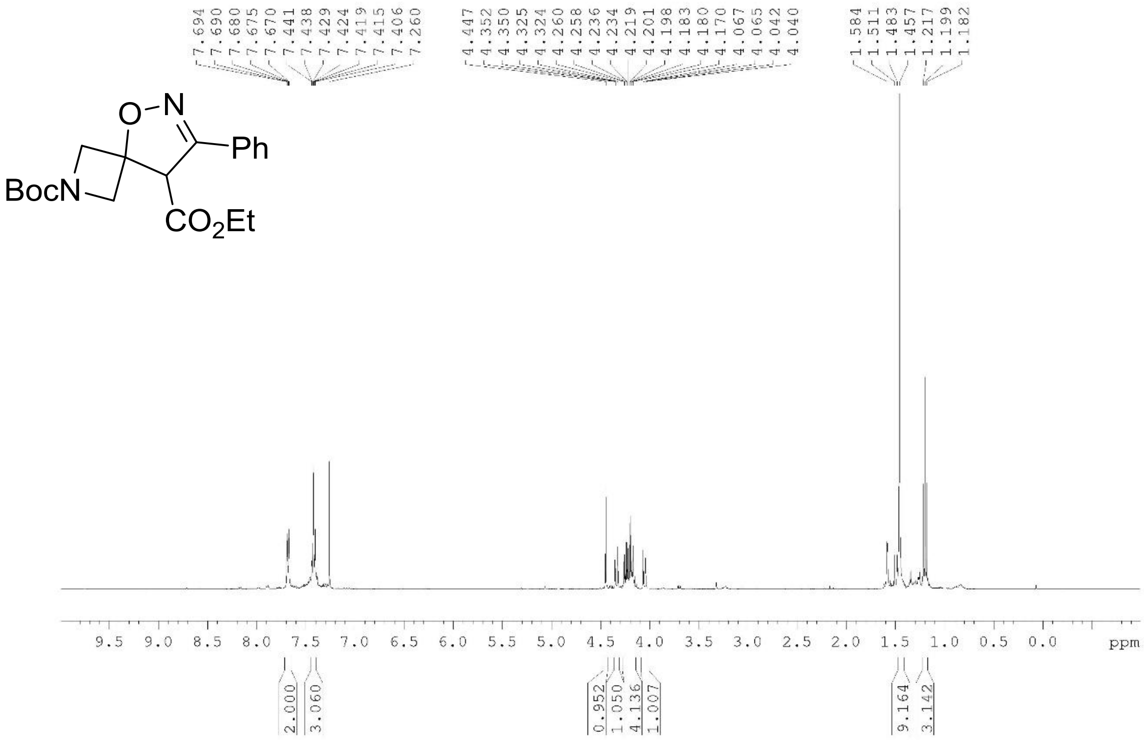

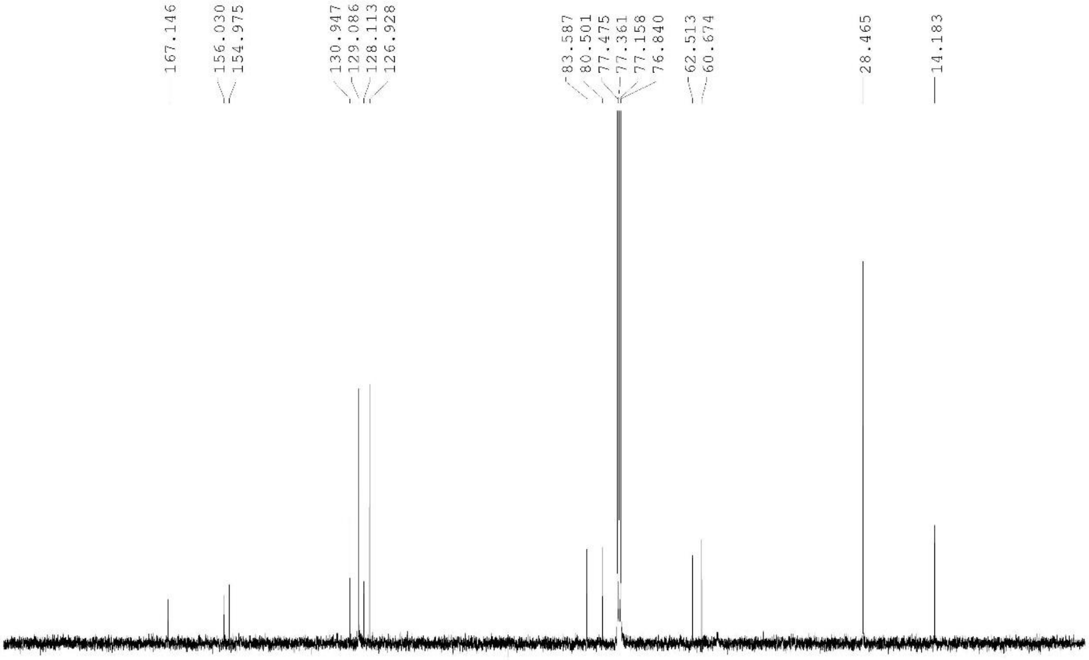

7-(tert-Butyl) 4-ethyl 3-phenyl-1-oxa-2,7-diazaspiro[4.4]non-2-ene-4,7-dicarboxylate (7c)

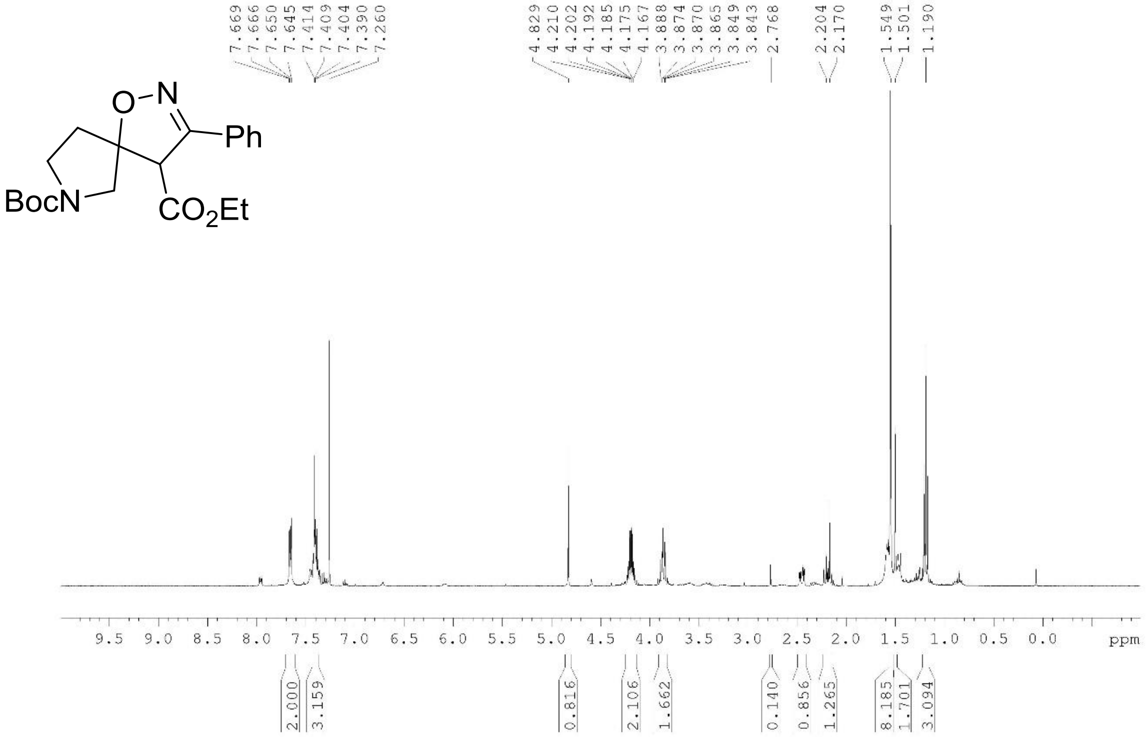

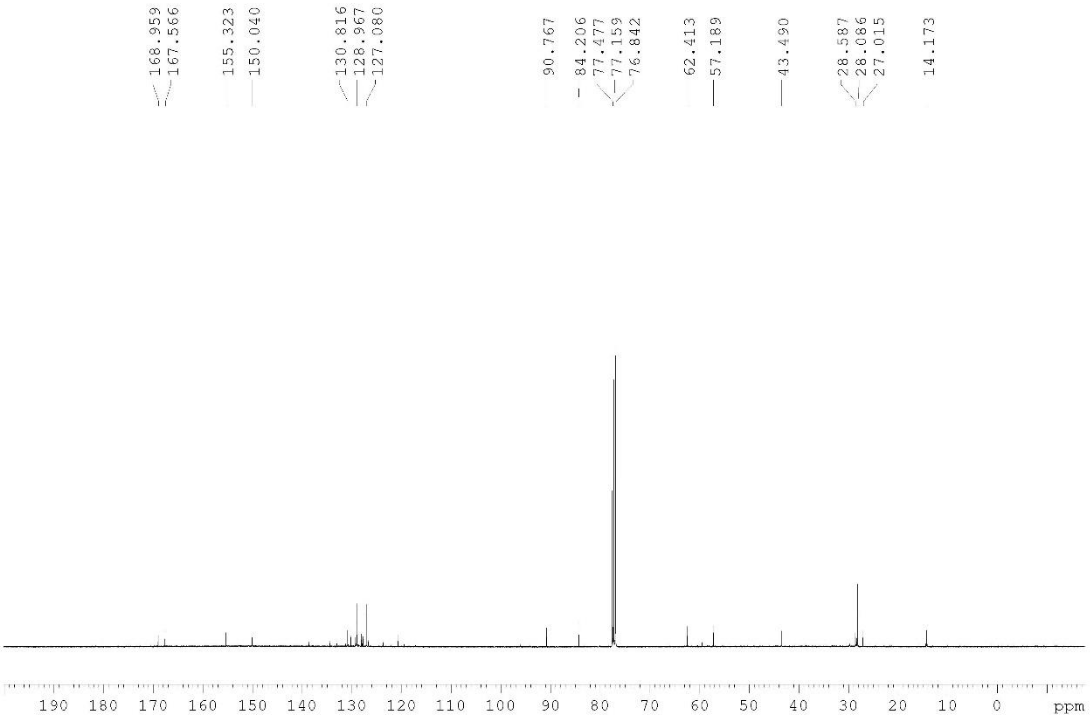

7-(tert-Butyl) 4-ethyl 3-phenyl-1-oxa-2,7-diazaspiro[4.5]dec-2-ene-4,7-dicarboxylate (7d)

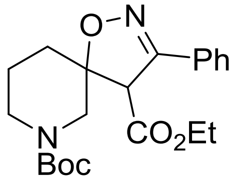

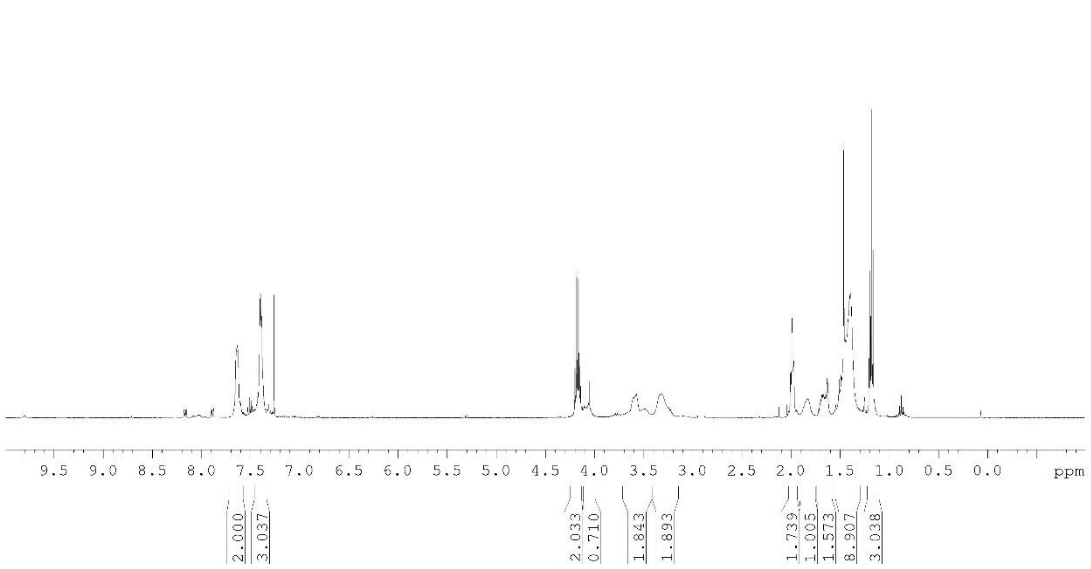

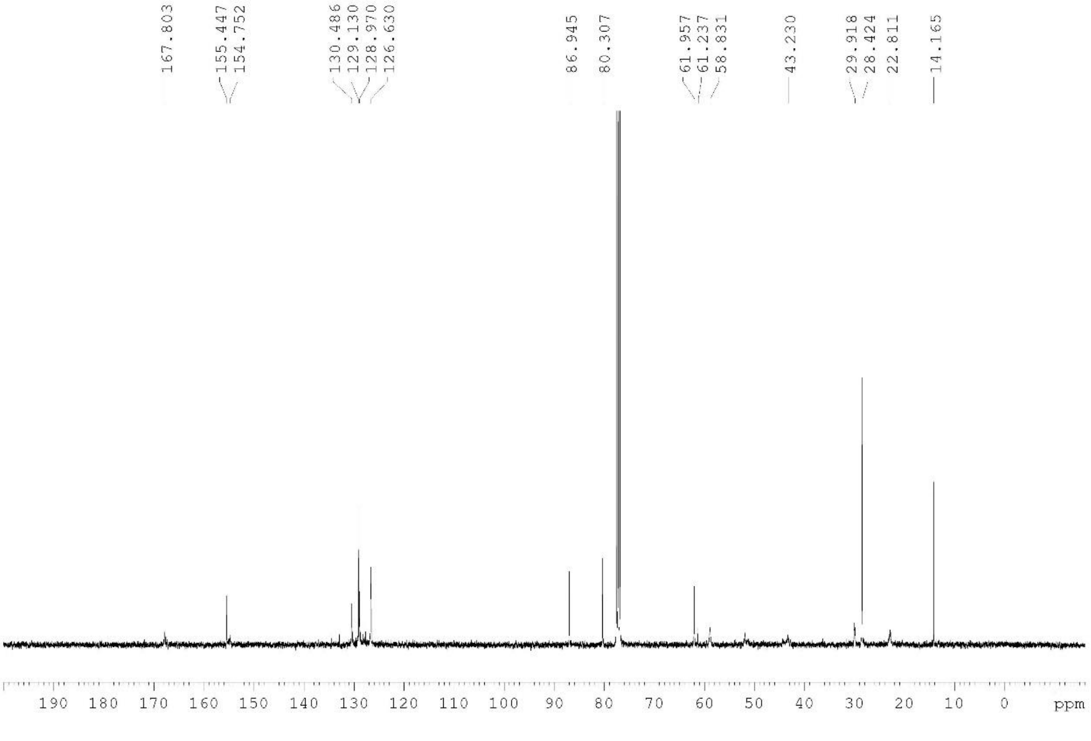

7-(tert-Butyl) 4-ethyl 3-phenyl-1-oxa-2,7-diazaspiro[4.6]undec-2-ene-4,7-dicarboxylate (7e)

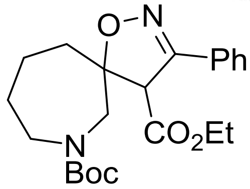

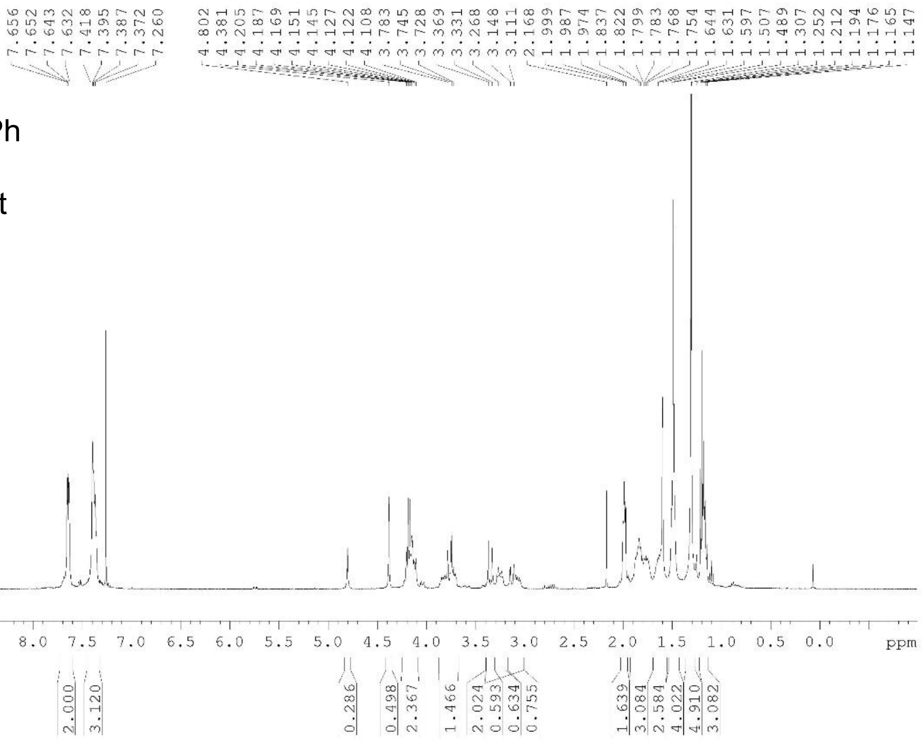

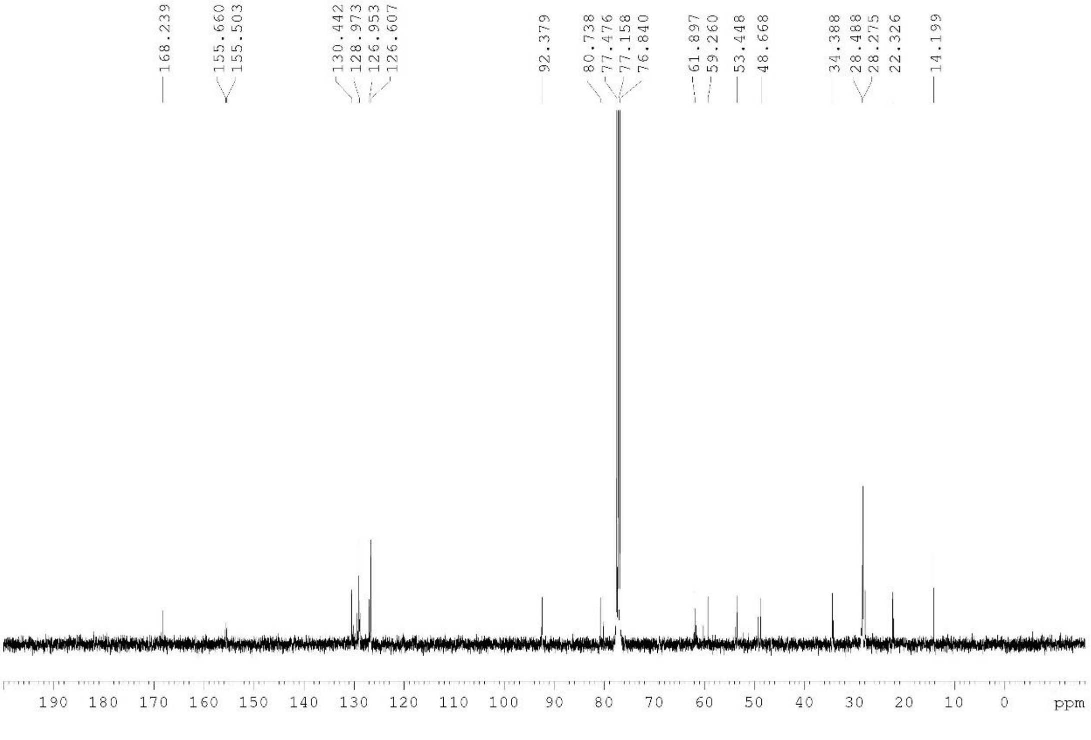

Ethyl 3-phenyl-1-oxa-2,8-diazaspiro[4.5]dec-2-ene-4-carboxylate (17a)

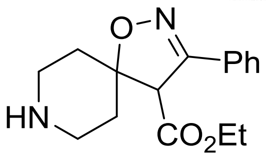

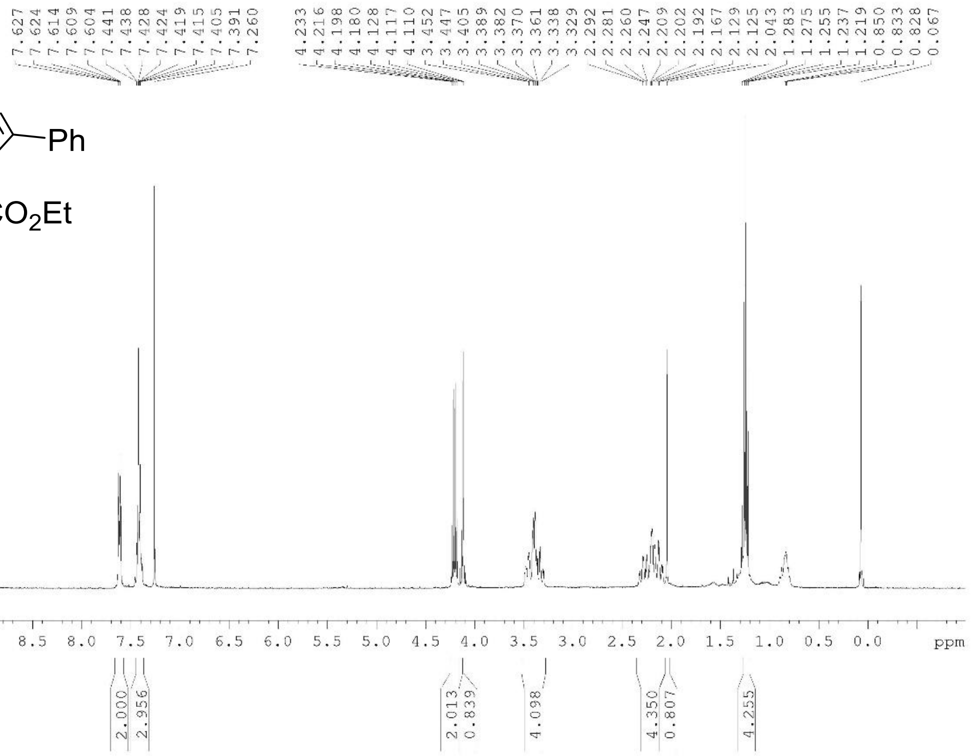

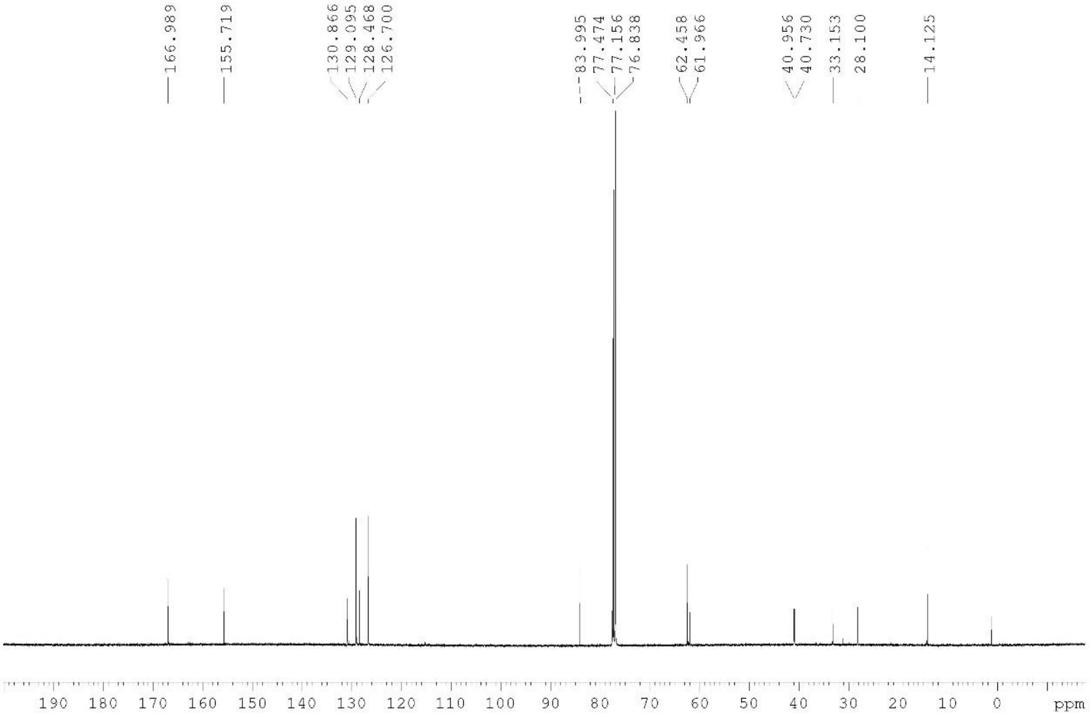

2-(tert-Butyl) 8-ethyl 7-benzyl-2,5,6,7-tetraazaspiro[3.4]oct-5-ene-2,8-dicarboxylate (8)

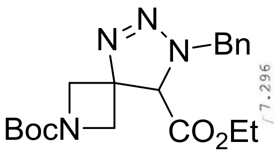

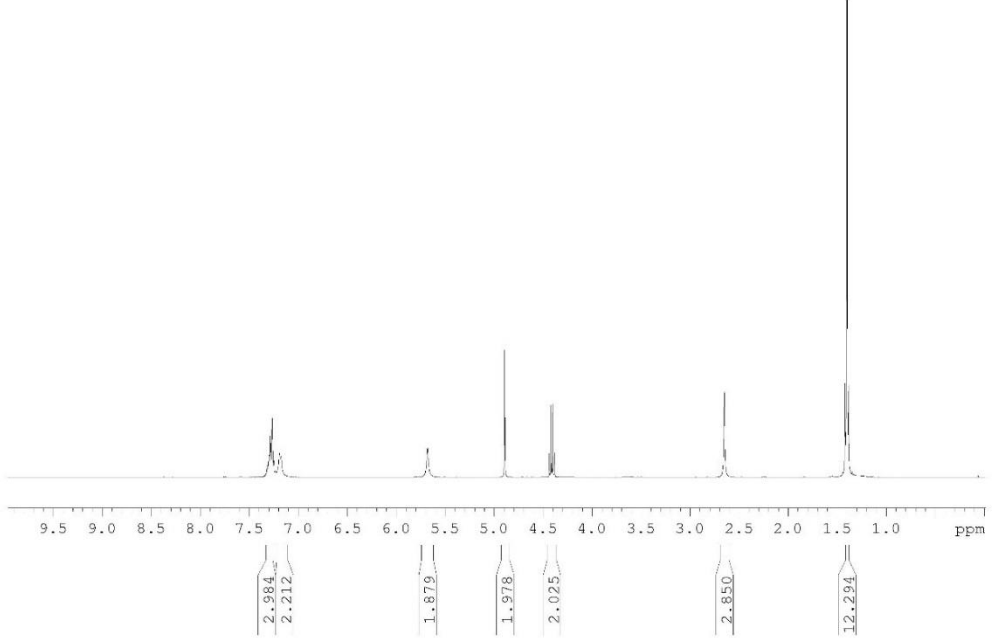

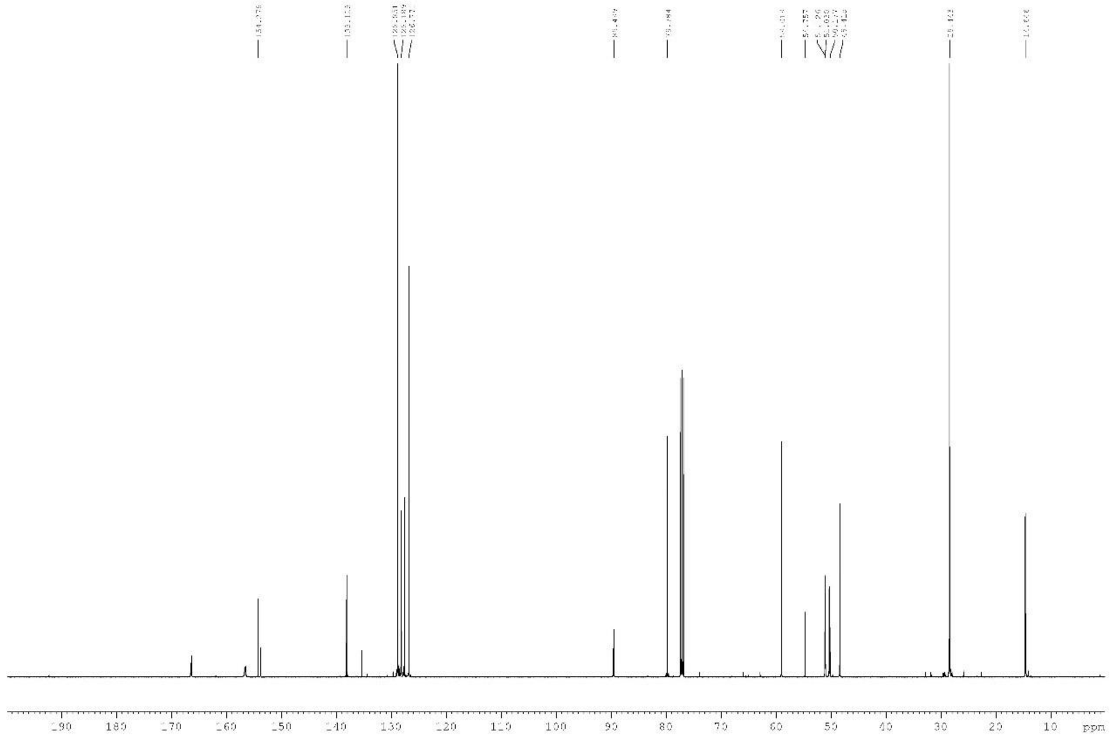

***tert*-Butyl 3-oxo-1,2,8-triazaspiro[4.5]decane-8-carboxylate (9)**

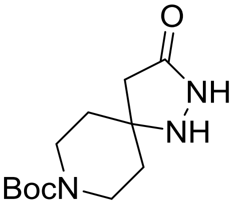

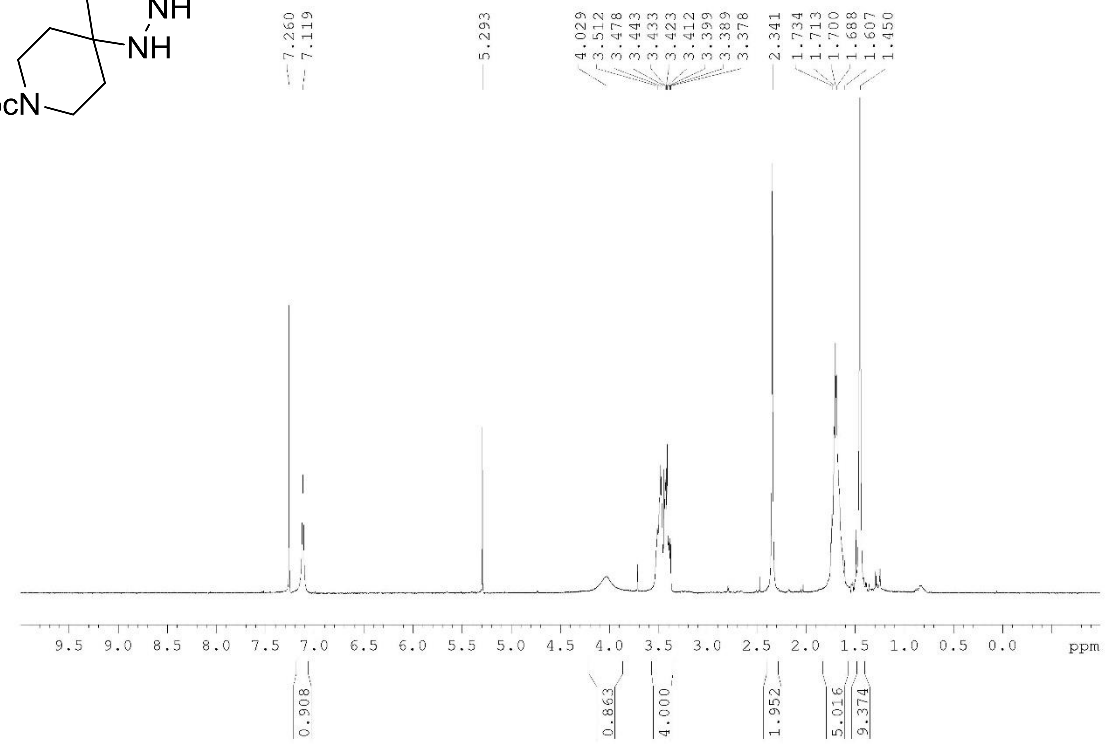

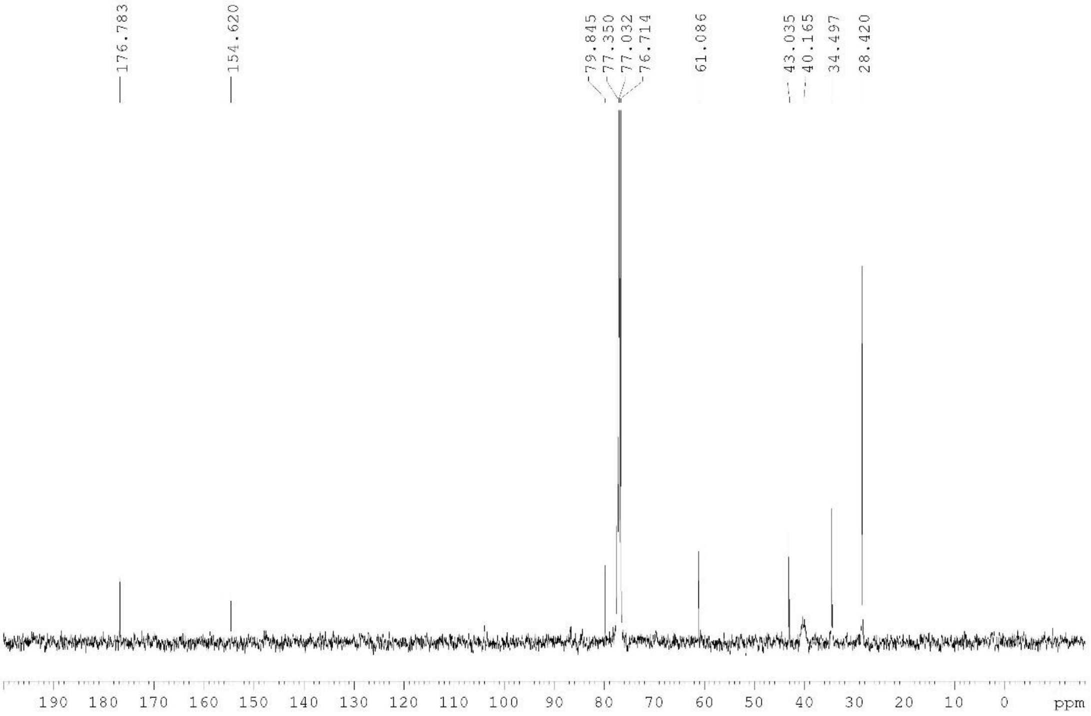

***tert*-Butyl 4-((4-methoxyphenyl)imino)piperidine-1-carboxylate (6)**

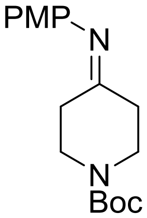

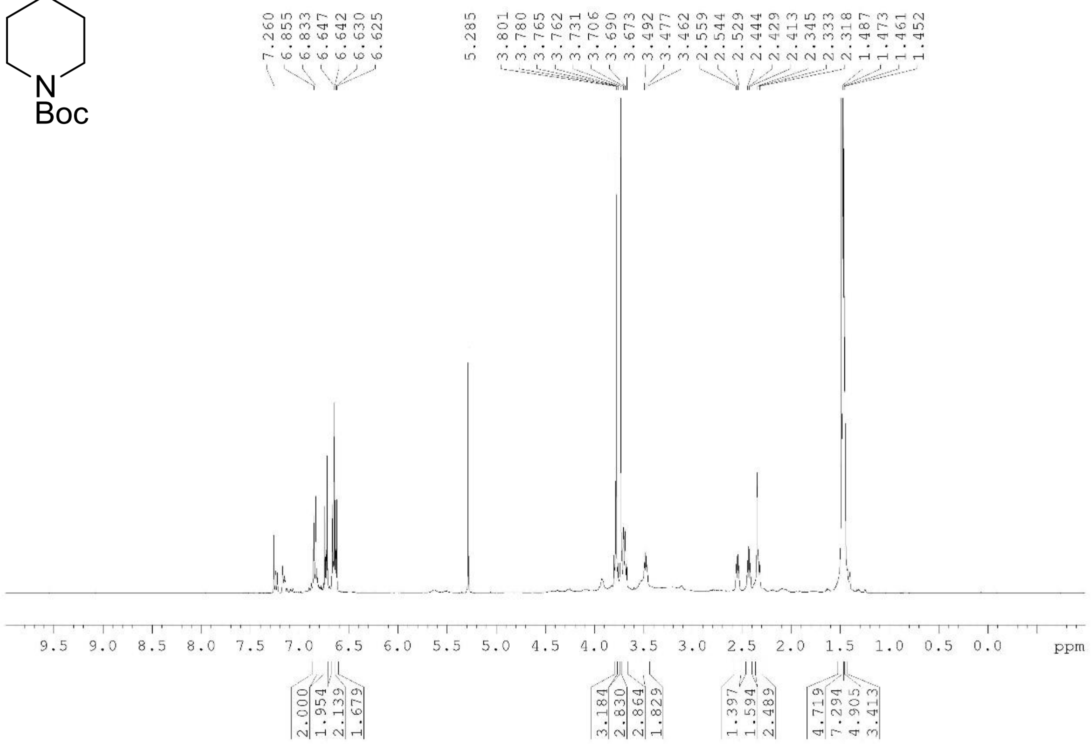

***tert*-Butyl 3-acetoxy-1-(4-methoxyphenyl)-2-oxo-1,7-diazaspiro[3.5]nonane-7-carboxylate (10)**

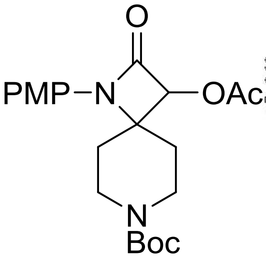

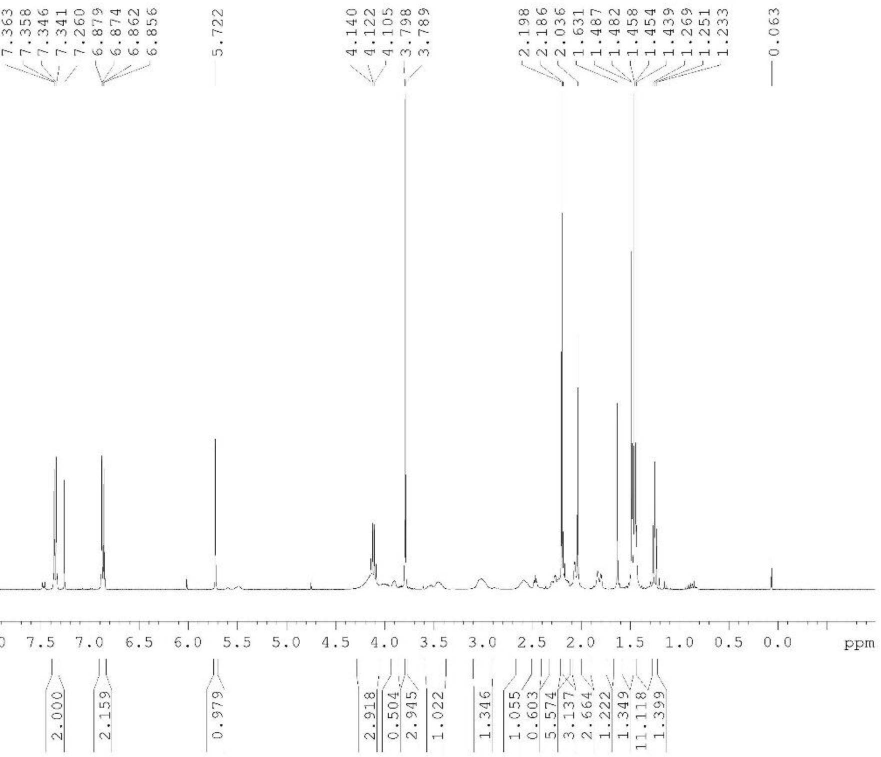

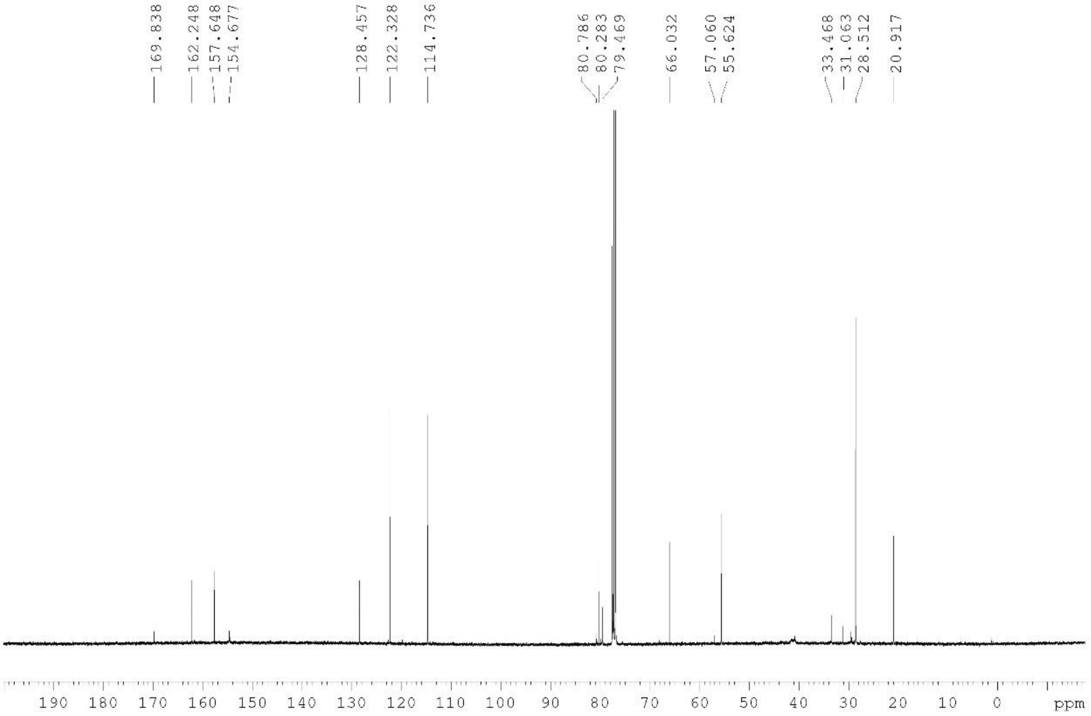

3. X-ray Crystallography

3-Phenyl-1,8-dioxa-2,6-diazaspiro[4.4]non-2-en-7-one (3)

Identification code	DS_B1_0017	
Empirical formula	$C_{11}H_{10}N_2O_3$	
Formula weight	218.2120 gmol^{-1}	
Temperature	180(2) K	
Wavelength	1.54178 Å	
Crystal system	Triclinic	
Space group	$P\bar{1}$(2)	
Unit cell dimensions	a = 5.3405(3) Å	α = 78.073(4)°
	b = 8.0452(5) Å	β = 77.997(4)°
	c = 12.0561(7) Å	γ = 81.581(4)°
Volume	492.828 Å^3	
Z	2	
Density (calculated)	1.470 gcm^{-3}	
Absorption coefficient	0.914 mm^{-1}	
F(000)	228	
Crystal size	0.180 × 0.040 × 0.020 mm^3	
Theta range for data colletion	3.81–66.58°	
Index ranges	–6 ≤ h ≤ 3, –9 ≤ k ≤ 9, –14 ≤ l ≤ 13	
Reflections collected	4038	
Independent reflections	1723	
Completeness to theta = 66.58°	98.7%	
Absorption correction	Multi-scan	
Max. and min. transmission	0.7528 and 0.6366	
Refinement method	Full-matrix least-squares on F^2	
Data/restraints/parameters	1723/0/149	
Goodness-of-fit F^2	1.036	
Final R indices [I > 2σ(I)]	R1 = 0.0482, wR2 = 0.1032	
R indices (all data)	R1 = 0.0790, wR2 = 0.1153	
Largest diff. peak and hole	0.224 and –0.250 eÅ^{-3}	

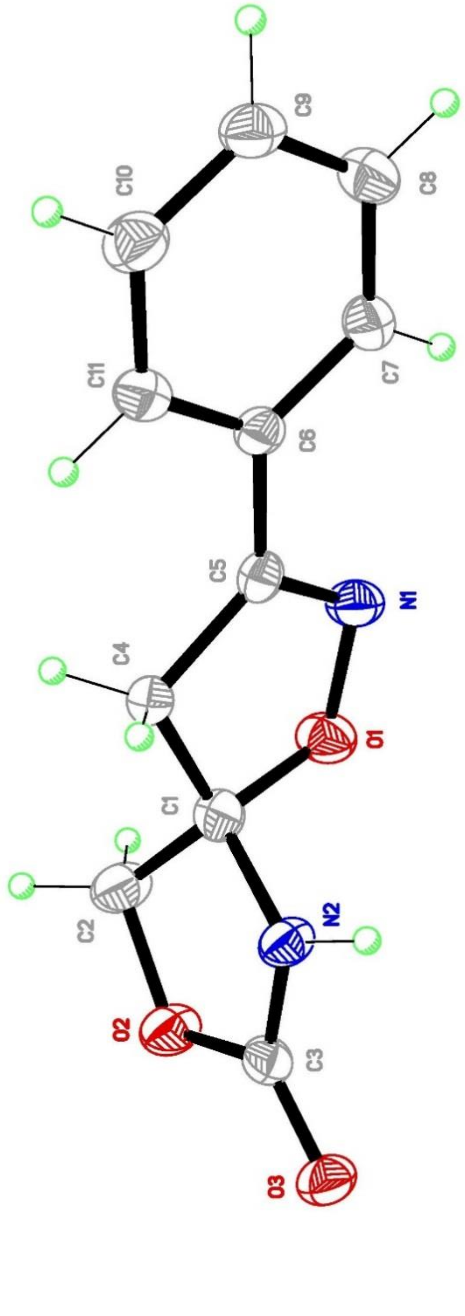
C9
C10
C8
C11
C7
C6
C5
N1
C4
O1
C1
N2
C2
O2
C3
O3
